# Teacher feedback VS AI-assisted peer feedback in L2 writing: A quasi-experimental study in a Chinese University

**DOI:** 10.1371/journal.pone.0345976

**Published:** 2026-06-29

**Authors:** Jinfeng Tang, Ping Li, Rong Luo

**Affiliations:** 1 School of International Communication, Wuhan Business University, Wuhan, Hubei, People’s Republic of China; 2 School of International Studies, Zhejiang University, Hangzhou, Zhejiang, People’s Republic of China; Golestan University, IRAN, ISLAMIC REPUBLIC OF

## Abstract

Despite the growing interest in AI-assisted peer feedback, few studies have systematically compared it with teacher feedback within a sociocultural framework. Consequently, the dynamic evolution of these two modes, their effects on micro-linguistic features, and the possibility of integrated feedback models remain largely unexplored. To address these gaps, this study, grounded in Vygotsky’s theories of scaffolding and the Zone of Proximal Development (ZPD), adopted an 8-week quasi-experimental design to conduct a multi-dimensional comparison of teacher feedback and AI-assisted peer feedback among 61 university students (244 writing texts). The innovation lies in revealing, for the first time from a “heterogeneous scaffolds” perspective, the essential differences and complementary mechanisms between the two feedback types within a sociocultural framework. The main findings are: (1) Teacher feedback demonstrated a characteristic of “comprehensive coverage and dynamic adjustment,” whereas AI-assisted peer feedback exhibited features of “sustained focus and efficient guidance.” (2) Teacher feedback produced significant short-term improvement but insufficient subsequent momentum, while AI-assisted peer feedback displayed a stable “learning-curve effect.” (3) AI-assisted peer feedback enhanced lexical diversity only in the initial task, and neither approach significantly improved syntactic complexity. In terms of practical contributions, this study proposes concrete pathways for optimizing teacher feedback and improving AI-assisted peer feedback, and constructs a hybrid “AI-Peer-Teacher” integrated feedback model, offering an actionable reform proposal for English writing instruction in application-oriented universities.

## Introduction

Writing, as a core product of Second Language Acquisition (SLA), directly reflects the internalization degree of a learner’s second language proficiency. In second language writing instruction, feedback serves as a crucial link between teaching and learning, facilitating learning by providing valuable insights into individual performance [1]. Over the past two decades, research on second language writing feedback has evolved from descriptive narratives to empirical investigations, shifting from a single methodological approach to more diverse and integrated research frameworks [2,3].

Early studies predominantly focused on the effectiveness of feedback delivered by teachers, peers, and automated writing evaluation (AWE) platforms such as Criterion [4–6]. With the emergence of generative AI tools represented by ChatGPT in recent years, AI-based feedback has become an innovative mediating tool in L2 writing instruction due to its strengths in content guidance and in-depth logical analysis [7–9]. Accordingly, research focus has further extended to emerging areas concerning AI-assisted feedback and large language model-enabled feedback [10–12]. Meanwhile, the research perspective has gradually shifted from primarily focusing on the external effects of feedback to emphasizing learners’ internal psychological processes, such as feedback engagement, feedback literacy, and emotional factors during the feedback process [13–15].

Extensive studies have confirmed the positive role of teacher feedback [16–18], but have also pointed out its limitations in terms of time cost and scalability [19,20]. Peer feedback, while alleviating teachers’ workload and promoting collaboration and critical thinking [21,22], is often constrained by students’ own language and assessment levels [23,24]. Recent studies have begun to compare AI-generated feedback with teacher feedback in L2 writing contexts. Findings indicate that while AI feedback can enhance learners’ revision quality, autonomy, and writing accuracy, teacher feedback continues to play a vital role in supporting content development, emotional engagement, and deeper feedback uptake [25,26].

While existing research has preliminarily validated the effectiveness of artificial intelligence tools in second language writing instruction, three significant research gaps remain: First, most comparative studies focus on static effect comparisons, lacking tracking of the dynamic evolution of the two feedback modes during intervention (e.g., how feedback priorities change throughout the learning process). Second, few studies examine the distinct impacts of the two feedback types on micro-linguistic features—such as lexical diversity and syntactic complexity—which serve as crucial indicators of language development. Third, current research predominantly favors one feedback mode while overlooking their complementary potential, with insufficient exploration into constructing integrated feedback models. More critically, systematic comparisons between teacher feedback and AI-assisted peer feedback (rather than standalone AI feedback) remain scarce within a unified theoretical framework. Compared to pure AI feedback, research on AI-assisted peer feedback mechanisms better highlights the interaction between technology and interpersonal dynamics, providing deeper insights into learner agency, collaborative learning patterns, and the social dynamics of feedback.

To address the aforementioned gaps, this study explicitly adopts Vygotsky’s (1978) Sociocultural Theory [27], particularly focusing on its two core concepts: “scaffolding” and the “Zone of Proximal Development” (ZPD). From this theoretical perspective, teacher feedback can be regarded as expert-provided dynamic scaffolding— characterized by its authority and context sensitivity, enabling adaptive support tailored to learners’ ZPD. In contrast, AI-assisted peer feedback functions as technology-mediated collaborative scaffolding, offering real-time assistance, standardized cognitive support, and peer-mediated interaction. These two scaffolding approaches exhibit distinct characteristics, operational mechanisms, and applicable boundaries, making it challenging for any single model to comprehensively address learners’ multidimensional ZPD. Therefore, this study employs an 8-week quasi-experimental design within a sociocultural framework to systematically compare the effects of teacher feedback versus AI-assisted peer feedback on second-language writing, with a focus on their dynamic evolution, impact trajectories on writing performance, and contributions to lexical diversity and syntactic complexity.The specific research questions are as follows:

RQ1: How do teacher feedback and AI-assisted peer feedback differ in terms of quantity and focus?

RQ2: How do teacher feedback and AI-assisted peer feedback differ in terms of their impact on students’ writing performance over time?

RQ3: What are the respective effects of teacher feedback and AI-assisted peer feedback on learners’ lexical diversity and syntactic complexity in L2 writing?

## Literature review

### Theoretical framework: Sociocultural theory and feedback in second language writing

Sociocultural Theory (SCT) provides a unified analytical framework for understanding the feedback mechanisms and operational logic in second language writing. Vygotsky (1978) emphasized that cognitive development does not occur in isolation but is constructed through social interaction, linguistic mediation, and contextual engagement [27]. The theory’s two core concepts—the Zone of Proximal Development (ZPD) and scaffolding—offer critical insights into feedback effectiveness: feedback essentially serves as an external scaffold whose value depends on its precise alignment with the learner’s cognitive potential within the ZPD and its gradual withdrawal at appropriate moments, thereby enabling learners to develop independent task completion skills [28]. In the context of second language writing, SCT further highlights that knowledge acquisition is a continuous process of “reconstruction, adjustment, and expansion” [29,30]. Effective written feedback must possess “dialogic” and “contextualized” characteristics—it is not merely a one-way transmission of information but rather a dynamic collaborative process of meaning construction between learners and experts (teachers or more capable peers) [28].

Notably, the traditional SCT framework exhibits a distinct binary structure in defining “support providers”: teachers are regarded as “expert scaffolds” with professional diagnostic capabilities, peers as “collaborative scaffolds,” while technical tools are merely classified as static “auxiliary intermediaries” [31]. With the emergence of generative AI, this classification faces theoretical challenges—AI can provide expert-level error-correction suggestions at the linguistic level and deliver instant dynamic responses at the interaction level, yet its scaffolding attributes remain inadequately theorized. This theoretical gap constitutes the starting point of this study.

### Definition of core concepts

To ensure clarity in the research concepts, this section provides clear definitions of the following key terms:

**Teacher Feedback (TF)**: Diagnostic information provided by second language writing instructors to learners in written or oral form, aimed at enhancing writing quality and writing process competencies, encompassing two dimensions: corrective feedback (CF) and content feedback (idea feedback). In this study, teacher feedback refers to the written comments and revision suggestions provided by instructors after evaluating learners’ compositions.
**Peer Feedback (PF)**: This refers to mutual evaluation activities among learners at similar language proficiency levels or comparable learning stages regarding the quality, structure, and content of written compositions. Its core mechanisms include meaning negotiation, collaborative interaction, and critical collaboration [21,22].
**AI Feedback (AIF)**: Refers to the diagnostic and revision suggestions generated by algorithms when learners input written texts into generative AI tools (e.g., ChatGPT). Unlike traditional automated writing evaluation (AWE), which only provides static scores or preset template feedback, generative AI feedback features dynamic dialogue, multi-round interactions, and personalized explanations [32].
**AI-Assisted Peer Feedback (AI-PF)**: The core construct of this study describes how learners, during peer review processes, utilize generative AI tools as collaborative enhancement mediators to diagnose and analyze peers’ compositions and generate feedback suggestions, facilitating face-to-face or digitally mediated collaborative feedback exchanges. This construct distinguishes itself from “pure AI feedback” (where learners use AI alone) and “pure peer feedback” (without AI involvement), emphasizing AI’s role as an enhancer of peer collaboration rather than a substitute for teachers.

### The evolution of research on second language writing feedback and its core challenges

Focusing on the core question of “which feedback mechanism can maximize the scaffolding effect,” existing research reveals three primary evolutionary trajectories and three fundamental dilemmas.

#### Teacher feedback: Effectiveness vs. Scalability.

In second language writing instruction, teachers’ corrective feedback (CF) serves as a crucial cognitive mediator. As early as the late 1990s, Ferris and Hedgcock (1998) refuted Truscott’s claim that written corrective feedback was ineffective [33,34]. Subsequent research has confirmed the effectiveness of corrective feedback in second language writing instruction [35,36], emphasizing the need for teachers to establish effective communication with students, provide constructive feedback, and implement reflective teaching practices [28,37]. Empirical studies demonstrate that direct corrective feedback from teachers significantly enhances learners’ linguistic accuracy and discourse structure skills [38,39].

However, the very strengths of teacher feedback also constitute its limitations. Although teacher feedback demonstrates high professionalism and diagnostic depth, it faces dual challenges of immediacy and scalability in large-scale teaching settings [40,41]. From the perspective of Sociocultural Theory, the scaffolding effect of teacher feedback relies on teachers’ accurate assessment of each learner’s Zone of Proximal Development (ZPD)—an assessment that requires substantial time and accumulated interaction, making it difficult to sustain as class sizes expand.

#### Peer feedback: Collaborative value vs. Capability limitations.

Over the past two decades, research on peer feedback has advanced rapidly. Scholars worldwide have confirmed its positive impact in second language writing instruction [42–44]. Studies demonstrate that through meaning negotiation and collaborative interaction, peer feedback effectively alleviates writing anxiety while enhancing critical thinking and self-directed learning abilities [45]. Within the Sociocultural Theory framework, peer feedback is regarded as a “collaborative scaffold among peers of similar proficiency,” whose value lies in facilitating knowledge reconstruction through social interaction.

However, the scaffolding effect of peer feedback is constrained by learners’ language proficiency and face-saving psychology [46,47]. Inadequate language skills result in inconsistent feedback quality, while sociocultural factors hinder the direct expression of critical opinions. To address these issues, scholars have proposed anonymous feedback mechanisms [48,49]; yet while anonymity alleviates face-saving concerns, it may also undermine the “dialogical” and “collaborative construction” characteristics emphasized by Sociocultural Theory.

#### Artificial intelligence feedback: Efficiency advantages vs. Limitations in depth.

With technological advancements, Automated Writing Evaluation (AWE) has been successfully integrated into second language writing instruction. Early web-based feedback platforms (e.g., Criterion, Pigai) have proven effective in identifying spelling, vocabulary, and grammatical errors, significantly reducing teachers’ grading time [50–52]. However, AWE systems typically only address surface-level language errors and exhibit limitations in assessing higher-level writing competencies such as content organization and logical coherence [53,54]. Consequently, numerous software developers advocate that automated writing evaluation should serve as an auxiliary tool rather than a substitute for teachers’ instructional responsibilities [55].

In recent years, the emergence of generative AI tools like ChatGPT has introduced a novel perspective on second-language writing feedback. These AI tools possess advanced text-generation capabilities and seamless human-computer interaction, enabling them to provide learners with precise suggestions and personalized revision guidance [56], thereby addressing the inherent timeliness limitations of teacher-based written feedback [12]. However, the accuracy and effectiveness of AI feedback still require further validation [9], and excessive reliance on artificial intelligence may impair learners’ critical thinking and reflective abilities [57,58].

#### From “substitution” to “collaboration”: A paradigm shift in integration approaches.

Existing literature exhibits systematic discrepancies in comparing the effectiveness of teacher feedback versus AI feedback. In terms of linguistic accuracy, AI feedback demonstrates significant advantages: Boudouaia et al. (2024) note that ChatGPT’s response speed for vocabulary correction and grammar refinement far surpasses that of teacher feedback [8], and it can provide multiple rounds of explanations for the same types of errors. However, regarding discourse structure and content development, teacher feedback remains irreplaceable—teachers can identify logical inconsistencies and inappropriate content choices based on a comprehensive understanding of learners’ writing intentions, whereas current AI feedback performance in this regard remains uncertain.

This divergence precisely validates the core tenet of the SCT framework: the effectiveness of feedback depends not only on its “accuracy,” but more crucially on the dynamic adaptation between scaffolding support and learners’ individual ZPD development. The strength of teacher feedback lies in its ability to enable diagnostic adjustments based on the learner’s individual cognitive state, whereas AI feedback excels in its immediacy and reproducibility. A key limitation in existing research is that most comparisons frame these approaches within an alternative logic (“Can AI replace teachers?”) rather than a complementary one (“Which combination of AI and feedback forms maximizes the scaffolding effect?”).

Against this backdrop, AI-assisted peer feedback has emerged as an integrated approach garnering increasing attention. Existing research demonstrates that AI-enhanced peer feedback significantly enhances learners’ engagement and writing quality [59], effectively addressing the quality shortcomings in traditional peer feedback caused by language proficiency disparities [60]. Compared to teacher feedback or purely AI-driven feedback, AI-assisted peer feedback offers unique collaborative reinforcement advantages: it preserves the social negotiation characteristics of peer interaction while leveraging AI to bridge feedback quality gaps arising from competency differences. However, feedback efficacy studies also indicate that although AI feedback provides abundant information, students may struggle to process excessive feedback content, leading to cognitive overload [61]. This finding suggests that the scaffolding effect of AI-assisted peer feedback is not automatic but depends on the alignment between AI intervention methods and peer collaboration structures.

#### Research gaps and study positioning.

Based on the aforementioned theoretical review, two specific and actionable research gaps exist in current studies:

Gap 1: The existing literature lacks a systematic comparison of the three scaffolding forms grounded in a unified theoretical framework.Existing literature primarily focuses on the binary contrast between teacher feedback and pure AI feedback [14], or explores the integrated application of AI tools with single feedback methods [59,62]. However, no study has treated AI-assisted peer feedback as an independent “social-technical composite scaffold” and examined its differences from teacher feedback in terms of scaffolding precision, dynamic adaptability, and cognitive load induction under a unified theoretical framework.
Gap 2: The “collaboration-enhancing mechanism” of AI-assisted peer feedback has not undergone sufficient empirical validation. Theoretically, AI-assisted peer feedback retains the social negotiation characteristics of peer interaction while leveraging AI to bridge the feedback quality gap caused by language proficiency differences; however, this hypothesis lacks rigorous experimental validation in second-language writing contexts. Existing studies predominantly employ descriptive or quasi-experimental designs, failing to adequately control for the independent and interactive effects of both “AI intervention” and “peer collaboration.”

Thus, the value of this study lies not only in verifying the impact of AI-assisted peer feedback on specific writing performance, but also in filling the aforementioned gap—by conducting a systematic comparative analysis of the scaffolding effects of teacher feedback versus AI-assisted peer feedback within the SCT framework, thereby providing empirical evidence for the effectiveness of “technology-mediated social collaboration scaffolding.”

## Research methodology

### Participants and research design

The study employed a pretest-posttest quasi-experimental design lasting 8 weeks. Participants were 61 second-year English majors from an application-oriented university in central China, with the original sample comprising six classes from the same grade. Prior to the study, all students from the six classes underwent a pretest. Based on the pretest scores and class learning performance, two classes were selected as the experimental (n = 30, receiving AI-assisted peer feedback) and control classes(n = 31, receiving traditional teacher feedback) to ensure intergroup balance and control for group effects.

The gender distribution was essentially identical between the two classes: the experimental class comprised 4 males and 26 females, while the control class consisted of 4 males and 27 females, with no significant differences between classes. All participants were sophomore university students who already possessed basic writing skills but had not received systematic training in argumentative writing, facilitating the observation of intervention effects. Regarding experience with AI tools, a pre-study questionnaire revealed that 12 participants (7 in the experimental class and 5 in the control class) had independently used ChatGPT or similar tools primarily for vocabulary queries or grammar checking, but none had received AI-assisted feedback in formal writing contexts.

Prior to participation, all students were informed of the purpose and procedures of the study. Written informed consent was obtained from all participants, indicating their voluntary agreement to participate and allowing the use of anonymized data for research purposes. Participant recruitment and consent collection were conducted between 14 March 2025 and 27 March 2025. The signed consent forms were collected and documented by the instructor, and all data were anonymized before analysis to ensure confidentiality.

The pre-test required both classes to write a 200-word argumentative essay, which was uploaded to the iWrite 2.0 platform, an online tool designed for assignment submission, basic evaluation, and scoring (without providing automated AI feedback). The resulting scores indicated that the control class scored 82.68 (SD = 5.48), while the experimental class scored 81.56 (SD = 5.65). The independent samples t-test indicated no significant difference between the two classes (t = 0.79, p = 0.415), confirming comparability of baseline levels. To ensure that the intervention effect was caused by the single variable of feedback mode, the teaching content, lesson periods, and instructor for both classes remained completely consistent throughout the study period.

### Feedback intervention

Both the experimental and control classes completed two writing tasks, producing two independent argumentative essays (approximately 200 words each). All participants submitted the first drafts via the iWrite 2.0 platform, for basic evaluation, and scoring.

The control class received written annotations and revision suggestions from the teacher based on a standardized five-dimensional feedback checklist (see Table 1). Students then revised their drafts according to the feedback and submitted second drafts (i.e., revised versions).

**Table 1:** Five-dimensional feedback checklist.

Number	Dimension	Specific content
1	Organizational structure	Problems in framework organization, paragraph logic, transitional coherence, etc.
2	Content theme expression	Problems in theme expression, clarity of argumentation, relevance to the topic, etc.
3	Grammar errors	Errors in tense, voice, clause usage, etc.
4	Vocabulary errors	Lexical omission, misuse, redundancy, etc.
5	Technical details	Errors in punctuation, capitalization, format, etc.

In the experimental class, students worked in pairs. Using the generative AI tool DeepSeek, they analyzed their partner’ s drafts according to the same five-dimensional checklist. Subsequently, based on the AI’ s suggestions, they discussed and negotiated to construct revision suggestions for their partner. Students then revised their own essays accordingly. Before the intervention, all students in this class received unified training on this collaborative feedback procedure. The teacher’ s role was limited to supervising the process and did not directly provide content feedback, thereby ensuring the scaffolding was primarily mediated by peers and AI.

**Fig 1:**
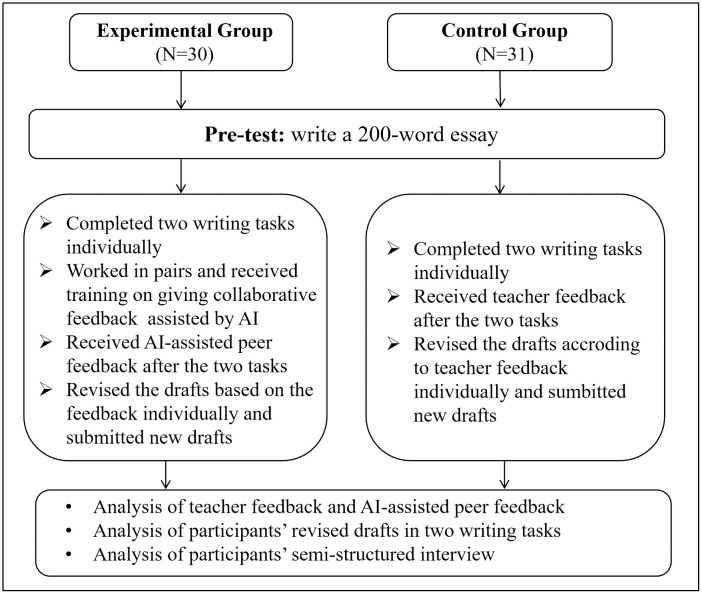
Research procedure.

The research procedure of this study is shown in the following Fig 1.

### Research instruments

To capture the process and effects of scaffolding, this study employed the following instruments:

Standardized Five-Dimensional Feedback Checklist: This checklist was adapted from Yang’s (2022) [63] Feedback Checklist and Orit Zeevy Solovey’s (2022) [64]Error Categories, aiming to systematically guide feedback focus and record feedback content. The checklist covers five dimensions: organizational structure, content and argumentation, grammar, vocabulary, and technical details (see Table 1, and Appendix for details), providing a structured operational framework for both scaffolding modes.

The adapted checklist underwent content validity evaluation by three experts with extensive experience in second language writing instruction (average teaching tenure exceeding 10 years). The experts independently assessed the representativeness, exclusivity, and operational definitions of each dimension and refined the wording of certain items based on feedback. The final version achieved a content validity index (CVI) of 0.92. Prior to the formal experiment, researchers conducted pilot evaluations using the checklist on 10 non-experimental sample essays, yielding Cronbach’s α coefficients for the five dimensions ranging from 0.78 to 0.86, demonstrating strong internal consistency reliability.

The Writing Assignment, Scoring, and Data Management Platform (iWrite 2.0): All writing assignments (including the pre-test and post-test timed writings) were conducted through the iWrite 2.0 platform [65]. This platform was used for the unified submission, scoring, and collection of essays, ensuring consistency in operational procedures. All essays were scored using a consistent rubric on the platform to quantify longitudinal changes in overall writing performance and track differences in improvement pathways resulting from various feedback modes.

Language Feature Analysis Tool (Coh-Metrix 3.0): To explore the deep impact of scaffolding on the internalization of language development, objective indicators of lexical diversity and syntactic complexity were calculated using Coh-Metrix 3.0 [66](see Table 2).

**Table 2:** Core indicators of Coh-metrix 3.0.

Dimension	Specific indicator	Description
Lexical diversity	Lexical diversity, type-token ratio, content word lemmas (LDTTRc), Lexical diversity, MTLD, all words (LDMTLDa), Lexical diversity, VOCD, all words（LDVOCDa)	Measuring lexical richness and semantic coverage
Syntactic complexity	Left embeddedness, words before main verb, mean (SYNLE), Number of modifiers per noun phrase, mean (SYNNP), Minimal Edit Distance, part of speech (SYNMEDpos), Sentence syntax similarity, all combinations, across paragraphs, mean (SYNSTRUTt)	Measuring diversity of syntax structure and modification richness

Specifically, lexical diversity is assessed through LDTTRc, LDMTLDa, and LDVOCDa. These indicators have been widely validated in the field of second language writing research and reliably reflect the lexical complexity and developmental level of texts [67,68]. Similarly, to precisely capture syntactic variety and modification richness, syntactic complexity is evaluated using four indicators: SYNLE, SYNNP, SYNMEDpos, and SYNSTRUT. These indicators constitute a mature framework for analyzing syntactic complexity in texts [69,70]. The empirically validated indicators above collectively serve as effective variables for revealing the extent of internalization in learners’ cognitive language development.

Statistical Analysis Software (SPSS 27.0): Used for all quantitative analyses, including descriptive statistics, independent samples t-tests, and repeated measures analysis of variance(ANOVA).

### Data collection and analysis

A total of 244 valid writing samples (from 61 students across experimental and control classes through two complete drafting-revision cycles) were collected and analyzed across three perspectives: feedback content, writing scores, lexical diversity and syntactic complexity.

Quantitative analysis proceeded in three directions. First, feedback content analysis was conducted, categorizing and counting the suggestion items according to the feedback checklist, and using independent samples t-tests to compare whether there were significant differences in the quantity of suggestion items. Second, score analysis was performed, using the scores from the iWrite platform as the dependent variable. Repeated measures ANOVA was employed to test the main effects of “feedback mode” (between-subjects factor) and “writing task” (time factor) on writing scores and their interaction effects, thereby revealing the different improvement pathways brought by the two feedback modes. Finally, language development analysis was conducted. Using the Coh-Metrix 3.0 text analysis tool, micro-level linguistic feature indicators of the essays were extracted. Similarly, repeated measures ANOVA was used to examine the impact patterns of the two feedback modes on various specific indicators of lexical diversity and syntactic complexity.

## Results

The analysis of this study revolves around three core questions.

### A comparison of the quantity and focus feature of teacher feedback and AI-assisted peer feedback

Quantitative analysis revealed significant differences between teacher feedback and AI-assisted peer feedback in focus, quantity, and dynamic changes.

#### Feedback quantity and distribution in the first writing task.

In the first task, the total amount of teacher feedback (316 items) was significantly higher than that of AI-assisted peer feedback (185 items). Independent samples t-tests indicated that this difference was particularly significant in the two dimensions of vocabulary errors and technical details: vocabulary errors (*t* (59) = −2.677, *P* = .010, Cohen’s d = 0.66), technical details (*t* (59) = −2.620, *P* = .012, Cohen’s d = 0.58) (see Table 3).

**Table 3:** Feedback comparison in the first writing task and results of independent sample t-tests.

Feedback dimension	Total feedback	Experimental class (n = 30)	Control class (n = 31)	*t*(59)	*P*	Cohen’s d	95% CI
		M (SD)	M (SD)				
Organizational structure	41	23 (0.76 ± 0.78)	18 (0.58 ± 0.95)	0.46	0.647	0.19	[-0.31, 0.67]
Content theme expression	106	46 (1.56 ± 2.18)	60 (1.92 ± 1.35)	0.48	0.631	0.17	[-1.38, 0.65]
Grammar errors	78	34 (1.12 ± 2.00)	44 (1.42 ± 1.50)	−0.612	0.543	0.15	[-1.29, 0.69]
Vocabulary errors	171	56 (1.88 ± 2.00)	115 (3.69 ± 2.75)	−2.677	0.010**	0.66	[-3.17, -0.45]
Technical details	105	26 (0.88 ± 1.42)	79 (2.54 ± 2.85)	−2.620	**0.012**	0.58	[-2.93, -0.38]
Total feedback	501	185 (6.16)	316 (10.19)		–	–	–

Note: The data in the table is formatted as “Total (M±SD)”. M = mean, SD = standard deviation, CI = confidence interval for mean difference.

#### Feedback quantity and distribution in the second writing task.

As the intervention progressed, the dynamic characteristics of the two feedback modes became further apparent. In the second task, the total amount of teacher feedback decreased (169 items), with the focus shifting to higher-order writing elements such as organizational structure and content/argumentation (total accounts for 46.15%). In contrast, the amount of suggestions in the content dimension for AI-assisted peer feedback sharply decreased, while feedback on technical details increased. Independent samples t-tests showed that in the content and argumentation dimension, the quantity of teacher feedback (M = 1.11, SD = 1.21) was significantly higher than that of the experimental class (M = 0.40, SD = 0.58), *t* (59)=−2.68, *P* = .010 (see Table 4).

**Table 4:** Feedback comparison in the second writing task and results of independent sample t-tests.

Feedback dimension	Total feedback (items)	Experimental class (n = 30)	Control class (n = 31)	*t*(59)	*P*	Cohen’s d	95% CI
		M (SD)	M (SD)				
Organizational structure	62	19 (0.64 ± 0.64)	43 (1.04 ± 0.16)	−1.93	0.060	0.49	[-0.81, 0.02]
Content theme expression	47	12 (0.40 ± 0.58)	35 (1.11 ± 1.21)	−2.68	0.010**	0.60	[-1.25, -0.17]
Grammar errors	35	16 (0.52 ± 0.87)	19 (0.62 ± 1.02)	−0.358	0.722	0.08	[-0.63, 0.44]
Vocabulary errors	89	37 (1.24 ± 1.05)	52 (1.69 ± 2.19)	−0.935	0.354	0.21	[-1.42, 0.52]
Technical details	56	36(1.20 ± 2.14)	20 (0.65 ± 1.29)	1.107	0.274	0.43	[-0.44, 1.54]
Total feedback	289	120 (4)	169 (5.45)				–

Note: M = mean, SD = standard deviation, CI = confidence interval for mean difference. *p < 0.05, ** p ≤ 0.01.

### Differential patterns of writing score improvement across two feedback modes

Repeated measures ANOVA revealed significant differences in the patterns of overall writing score improvement between the two feedback modes. Although both feedback modes led to statistically significant score gains, their effects over time differed significantly.

After the first writing task, the score improvement for the teacher feedback class(4.06 points) was slightly higher than that of the AI-assisted peer feedback class (3.87 points). However, in the second task, the improvement for the teacher feedback class decreased significantly (a drop of 32.8%), while that of the AI-assisted peer feedback class remained stable (3.89 points), ultimately surpassing the average score of the control class (see Table 5).

**Table 5:** Improvement in writing performance: within-group changes and between-group comparison.

Group	Writing session（N）	Draft score M(SD)	Revision score M(SD)	Within group improvement				Between-group comparison of gain scores		Cohen’s d
				Mean Difference [95% CI]	*t*(df)	*P*	Cohen’s d	Mean Diff. [95% CI]	*t*(df)	
Control class	First(31)	82.72(5.45)	86.78(4.15)	4.06 [2.38,5.74]	4.977(25)	<.001	0.97	–	–	–
	Second(31)	85.62(4.80)	88.35(5.52)	2.73[1.46,3.99]	4.453(25)	<.001	0.87	–	–	–
Experimental class	First（30）	81.24(5.85)	85.11(7.02)	3.87[1.38,6.37]	4.453(24)	.004	0.64	−0.19[−3.10, 2.72]	−0.131(59)	−0.037
	Second(30)	85.93(5.11)	89.82(5.45)	3.89[1.98,5.80]	4.194(24)	<.001	0.84	1.16[−1.06, 3.38]	1.048(59)	0.294

Note: M = mean, SD = standard deviation, CI = confidence interval for mean difference. Difference indicates the difference.

### Analysis of lexical diversity and syntactic complexity

To delve into the deep impact of the two scaffolding modes on the internalization of language development, this study analyzed lexical diversity and syntactic complexity. The specific inter-group differences are detailed in Table 6.

**Table 6:** Differences in lexical diversity and syntactic complexity between experimental class and control class.

Dimension	Indicators	Text versions	Control class (n = 31)	Experimental class (n = 30)	*t*	*P*	Cohen’s d
Lexical diversity	LDTTRc	Draft 1	0.71 ± 0.06	0.8 ± 0.07	−4.854	*** < 0.001	**−1.360**
		Revision 1	0.71 ± 0.06	0.79 ± 0.07	−4.664	*** < 0.001	**−1.306**
		Draft 2	0.75 ± 0.08	0.81 ± 0.06	−2.887	**0.006	**−0.809**
		Revision 2	0.76 ± 0.07	0.8 ± 0.06	−2.416	*0.019	−0.677
	LDMTLDa	Draft 1	78.95 ± 21.61	102.16 ± 29.68	−3.202	**0.002	**−0.897**
		Revision 1	78.46 ± 18.59	97.8 ± 19.69	−3.608	***0.001	**−1.011**
		Draft 2	93.53 ± 27.57	105.09 ± 27.11	−1.508	0.138	−0.422
		Revision 2	96.27 ± 31.55	106.69 ± 29.51	−1.217	0.229	−0.341
	LDVOCDa	Draft 1	75.15 ± 16.03	94.73 ± 27.13	−3.122	**0.003	**−0.883**
		Revision 1	77.37 ± 16.15	91.73 ± 19.72	−2.852	**0.006	−0.799
		Draft 2	87.87 ± 24.13	92.18 ± 19.21	−0.703	0.485	−0.197
		Revision 2	90.81 ± 25.32	92.13 ± 20.51	−0.203	0.84	−0.057
Syntactic complexity	SYNLE	Draft 1	5.88 ± 2.11	5.32 ± 2.02	0.972	0.336	0.272
		Revision 1	6.08 ± 2.13	5.99 ± 2.41	0.132	0.895	0.037
		Draft 2	5.88 ± 1.79	5.09 ± 1.27	1.819	0.075	0.509
		Revision 2	5.81 ± 1.76	5.31 ± 1.19	1.184	0.242	0.332
	SYNNP	Draft 1	0.96 ± 0.15	0.99 ± 0.17	−0.623	0.536	−0.174
		Revision 1	0.97 ± 0.15	1 ± 0.14	−0.802	0.426	−0.225
		Draft 2	0.94 ± 0.12	1.06 ± 0.17	−2.803	**0.007	−0.785
		Revision 2	0.99 ± 0.15	1.05 ± 0.16	−1.312	0.196	−0.367
	SYNMEDpos	Draft 1	0.63 ± 0.05	0.65 ± 0.05	−1.495	0.141	−0.419
		Revision 1	0.63 ± 0.03	0.65 ± 0.04	−1.504	0.139	−0.421
		Draft 2	0.63 ± 0.04	0.63 ± 0.05	0.072	0.943	0.02
		Revision 2	0.63 ± 0.04	0.62 ± 0.05	0.748	0.458	0.21
	SYNSTRUTt	Draft 1	0.1 ± 0.02	0.12 ± 0.05	−2.191	*0.035	−0.621
		Revision 1	0.1 ± 0.02	0.11 ± 0.04	−1.047	0.300	−0.293
		Draft 2	0.13 ± 0.03	0.12 ± 0.03	0.434	0.666	0.121
		Revision 2	0.13 ± 0.03	0.12 ± 0.03	1.089	0.281	0.305

Notes: p* < 0.05, p* < 0.01, p** < 0.001.

Cohen’s d: 0.2 = small effect, 0.5 = moderate effect, 0.8 = large effect.

#### Analysis of lexical diversity.

**Fig 2:**
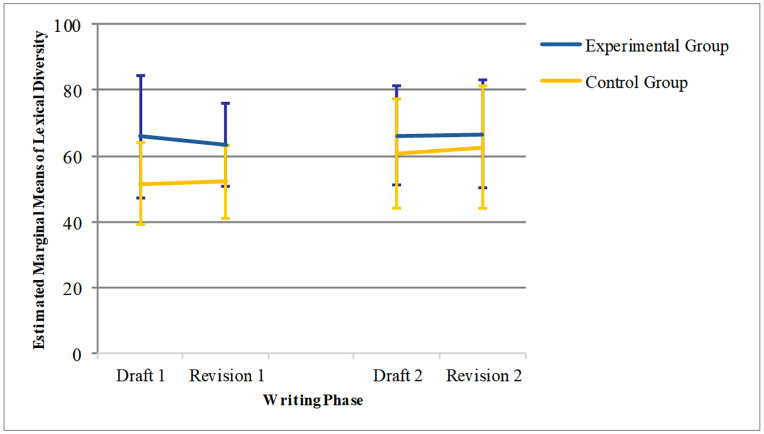
Evolution of lexical diversity.

Lexical diversity analysis aimed to examine whether the feedback mode could promote students to use richer and more diverse vocabulary. Regarding lexical diversity, the effect of feedback mode showed significant periodic characteristics. The evolution trend of lexical diversity before and after two writing tasks is shown in Fig 2.

Descriptive statistics revealed that, in the first writing task, the AI-assisted peer feedback class performed significantly better in lexical diversity than the teacher feedback class (F(1,59)=12.288, p < .001). However, this advantage disappeared in the second task, with the main effect of group no longer being significant (F(1,59)=1.084, p = .303), and the performance of the two groups converged(see Table 7).

**Table 7:** Results of mixed ANOVA for lexical diversity and descriptive statistics.

Writing task	Group	n	Draft (M ± SD)	Revision (M ± SD)	Time Main Effect (ηp²)	Group Main Effect (ηp²)	Interaction Effect (ηp²)
**First task**	Experimental class	30	65.89 ± 18.56	63.44 ± 12.65	*F* = 0.484	*F* = 12.288***	*F* = 1.249
	Control class	31	51.60 ± 12.27	52.18 ± 10.99	(*p* = .490)	(*p < .001)	(*p* = .269)
					ηp² = .010	**ηp² = .200**	ηp² = .025
**Second** **task**	Experimental class	30	66.02 ± 15.03	66.54 ± 16.35	*F* = 0.615	*F* = 1.084	*F* = 0.202
	Control class	31	60.72 ± 16.70	62.61 ± 18.62	(*p* = .437)	(*p* = .303)	(*p* = .655)
					ηp² = .012	ηp² = .022	ηp² = .004

Note: *p < .001; ηp² is the partial eta-squared effect size (small effect ≥ .01, moderate effect ≥ .06, large effect ≥ .14).

#### Analysis of syntactic complexity.

**Fig 3:**
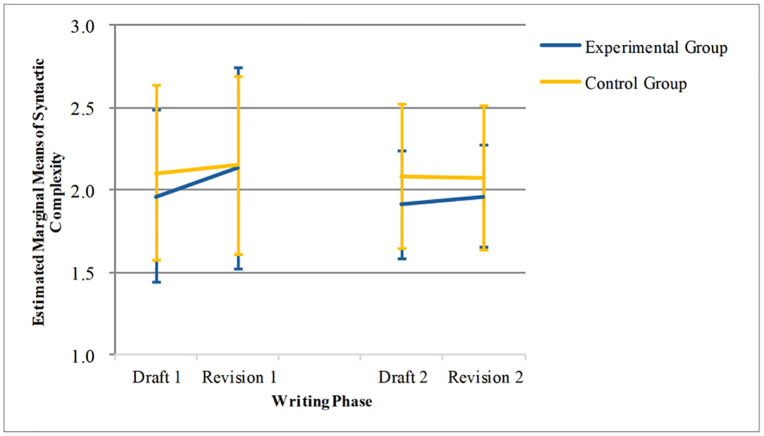
Evolution of syntactic complexity.

Syntactic complexity analysis aimed to examine whether the feedback mode could promote students to produce sentences with more complex structures and richer modifications. Regarding syntactic complexity, the enhancing effect of both feedback modes was limited and did not show differential advantages. The figure below illustrates the evolution in syntactic complexity before and after feedback for two writing tasks (see Fig 3).

According to descriptive statistics, in the first writing task, a significant main effect of time was found (F = 6.184, p = .016), indicating that feedback and revision behavior brought about a general improvement in syntactic complexity (composite mean increased from 2.03 to 2.14). However, neither the main effect of group nor the interaction effect was significant (*p* > .05), suggesting that this improvement was not driven by a specific feedback mode. In the second task, all effects were non-significant (time main effect: F(1,59)=0.234, *p* = .630; group main effect: F(1,59)=1.991, *p* = .165; interaction effect: F(1,59)=0.417, *p* = .522), indicating that syntactic complexity did not undergo systematic changes due to revision or different feedback modes(see Table 8).

**Table 8:** Results of mixed ANOVA for syntactic complexity and descriptive statistics.

Writing task	Group	n	Draft (M ± SD)	Revision (M ± SD)	Time main effect (ηp²)	Group main effect (ηp²)	Interaction effect (ηp²)
**First task**	Experimental class	30	1.96 ± 0.52	2.13 ± 0.61	F = 6.184*	F = 0.242	F = 1.905
	Control class	31	2.10 ± 0.53	2.15 ± 0.54	(*p* = .016)	(*p* = .625)	(*p* = .174)
					**ηp² = .112**	ηp² = .005	ηp² = .037
**Second task**	Experimental class	30	1.91 ± 0.33	1.96 ± 0.31	F = 0.234	F = 1.991	F = 0.417
	Control class	31	2.08 ± 0.44	2.07 ± 0.44	(*p* = .630)	(*p* = .165)	(*p* = .522)
					ηp² = .005	ηp² = .039	ηp² = .008

Note: *p < .001; ηp² is the partial eta-squared effect size (small effect ≥ .01, moderate effect ≥ .06, large effect ≥ .14).

## Discussion

Based on quantitative data analysis, this study systematically examines the mechanisms by which teacher feedback and AI-assisted peer feedback function as distinct forms of instructional scaffolding in second language writing instruction.

Specifically, it discusses differences in feedback focus and distribution, compares their effects on writing score development over time, and explores their influence on micro-linguistic features, including lexical diversity and syntactic complexity. Grounded in Sociocultural Theory, the findings illuminate how expert-driven and technologically mediated scaffolds differ in their modes of support and patterns of influence in L2 writing instruction.

### Differences in feedback focus characteristics and scaffolding nature

The research findings reveal key distinctions between the two feedback types across five dimensions. In the first writing task, teacher feedback significantly outperformed AI-assisted peer feedback, particularly in vocabulary errors and technical details. In the second task, teacher feedback shifted focus to higher-order writing elements like structural organization and content argumentation, while AI-assisted peer feedback reduced content-focused suggestions and increased technical detail guidance. Overall, teacher feedback demonstrated a dynamic evolution from comprehensive error correction to higher-order element guidance, whereas AI-assisted peer feedback maintained continuous support for operational aspects like technical details.

Teacher feedback demonstrates the high situational sensitivity and dynamic adaptability of an “expert dynamic scaffold.” In the initial intervention phase, the teacher, acting as an “authoritative error corrector,” invested significant effort in correcting basic language forms such as vocabulary and technical details. As students’ foundational issues improved, the feedback focus shifted toward higher-order dimensions like organizational structure and content/argumentation. This process reflects the adaptive support provided by an expert based on continuous diagnosis of the learner’s Zone of Proximal Development [30,71]. However, this deeply personalized support comes with high time costs and limited scalability [40,41].

In contrast, AI-assisted peer feedback embodies the characteristics of a “technologically mediated and socially collaborative scaffold” that is “core-focused and steadily guided” [60]. This mode consistently anchors feedback in the dimensions of content and structure. Although the total amount of feedback is lower, its focus is stronger. The sharp decrease in feedback quantity in the “content and theme expression” dimension for the experimental class indicates the significant efficiency of this scaffold in enhancing core conceptualization capabilities. However, its limitation lies in insufficient sustained attention to language form issues, leading to an increase rather than a decrease in feedback on “technical details” for the experimental class. This reflects a blind spot in current generative AI when dealing with language form problems requiring deep contextualized judgment. Furthermore, students find it difficult to overcome this blind spot when using AI for peer review, thus failing to continuously reduce such errors.

### Differences in writing performance improvement pathways

The pathways of language development is not linear and smooth but is full of dynamic fluctuations and imbalances [72]. The differences in writing score improvement trajectories further confirm the different pathways of the two scaffolds in promoting learning internalization. Teacher feedback achieved a greater immediate improvement in the first task, benefiting from its “comprehensive coverage” scaffold that could quickly and authoritatively correct a large number of overt errors, significantly narrowing the gap between student performance and the target in the short term. However, this feedback mode centered on “authoritative error correction” may encourage students to adopt relatively passive, error-avoidance-oriented revision strategies. More importantly, research points out that if teacher feedback overemphasizes error correction while neglecting to provide balanced, high-quality praise for learners’ efforts and progress, it may not only increase students’ psychological pressure but also weaken their learning motivation and willingness to take cognitive risks, thereby limiting the motivational role feedback should play [73,74]. When the learning challenge shifts from “error correction” to higher-order breakthroughs like “structural optimization” and “content deepening,” its improvement significantly decreased in the second task, indicating a progress bottleneck. This suggests that unidirectional, highly authority-dependent expert feedback has limitations in stimulating students’ autonomous exploration, risk-taking, and engagement in sustained deep learning, potentially making it difficult to effectively support students in crossing higher-level ZPDs [37, 72].

In stark contrast, the AI-assisted peer feedback class showed a clear “learning curve effect.” Its improvement remained stable between the two tasks, and it achieved an average score surpassing the control class in the second task. This indicates that AI-assisted peer feedback constructs a “technology-social” collaborative exploration system. As students’ proficiency with the tool and their collaborative synergy increase, their meta-cognitive and collaborative abilities—to independently identify problems, evaluate AI suggestions, and formulate revision plans through social negotiation—are continuously activated and strengthened [75]. The essence of this collaborative mode is that it gradually internalizes the externally provided mediational tool (AI) [76]and the social interaction pattern (peer negotiation) into individual cognitive tools, thereby releasing more enduring and autonomous progressive momentum [77]. Research has found that learners are more inclined to attempt L2 linguistic features in collaborative activities than in teacher-dominated situations [78]. This mode more closely approximates the ideal path described by Vygotsky, where psychological functions are internalized through socially mediated activities.

### Developmental characteristics of lexical diversity and syntactic complexity

The analysis of micro-linguistic features reveals the different impact of the two scaffolds on the development of different levels of language ability. Regarding lexical diversity, AI-assisted peer feedback showed an immediate advantage only in the first task. This may be due to the fact that, while offering content suggestions, AI provides abundant lexical choices or expressions, thereby furnishing students with an immediate “resource scaffold” for vocabulary [79,80] However, this advantage has not been internalized into a stable lexical generation strategy for the students.

Regarding syntactic complexity, neither feedback mode showed significant advantages. Syntactic ability involves the automated use of complex structures like clause embedding and phrasal modification, representing a deeper layer of language proficiency [81]. The results of this study indicate that both teacher feedback and AI-assisted peer feedback have very limited promoting effects on it. This strongly suggests that the development of syntactic ability may reside within a higher-order ZPD, whose breakthrough requires more systematic, sustained, and specialized instructional interventions (such as targeted sentence pattern training, explicit syntactic awareness cultivation, etc.) than general writing feedback [82]. This finding highlights the hierarchical nature of language ability development and the issue of matching between scaffolding types and levels of language development [83]. Specifically, while teacher feedback exhibits dynamic adaptability, within the limited class hours, its focus naturally shifts from basic linguistic forms to content and structure, making it difficult to maintain long-term concentration on syntax alone. Although AI-assisted peer feedback provides immediate suggestions, its general language models have not yet been capable of systematically identifying learners’ syntactic weaknesses and offering targeted training. Therefore, enhancing syntactic complexity may require an intervention period longer than the eight weeks in this study, along with the design of specialized intermediary strategies tailored to syntactic development rather than relying solely on general writing feedback.

In summary, within the Sociocultural Theory framework, this study confirms that teacher feedback and AI-assisted peer feedback are “heterogeneous scaffolds” with different natures and complementary advantages. No single mode can comprehensively address the multidimensional challenges of L2 writing ability development. Therefore, the results of this study strongly support constructing a multi-level, dynamically complementary “AI-Peer-Teacher” hybrid integrated feedback model to form a more complete network of mediational activity.

## Implications and suggestions

This study proposes the following systematic implications and suggestions for feedback practices in English writing instruction at application-oriented universities.

### Optimization pathways for traditional teacher feedback

Given the reduced volume of teacher feedback in the second phase but its shift toward higher-order elements (accounting for 46.15% of total feedback), the optimization strategy involves: First, implementing AI-assisted screening. Teachers can leverage AI tools to process high-frequency, overt language form issues (e.g., obvious grammar and spelling errors) in bulk, freeing up valuable cognitive and time resources from “comprehensive error correction” to more accurately diagnose and address personalized higher-order problems requiring deep contextual understanding. Second, adopting dynamic, competency-oriented feedback. Teachers should adjust feedback focus based on students’ learning progress, gradually reducing intervention in foundational dimensions while increasing guidance on higher-order dimensions, thereby evolving their role from “error correctors” to “thinking facilitators.”

### Improvement strategies for AI-assisted peer feedback

To address the phenomenon where experimental class experienced a sharp reduction in content dimension suggestions during the second task while technical detail feedback increased, improvement strategies should include: First, designing structured guidance and verification mechanisms by embedding specific prompts for basic language forms and peer review verification steps into the AI-assisted feedback process, ensuring continuous correction of fundamental issues. Second, enhancing teachers’ precise supervision and process intervention. Teachers should assume the roles of “supervisors” and “supporters,” conducting sampling reviews of feedback quality and depth of social negotiation, while providing timely personalized interventions for struggling groups. Additionally, specialized training on using intermediary tools should be strengthened. Regarding student feedback issues (such as AI feedback being “mechanically rigid” or “inadequate prompt alignment”), training should be implemented to improve students’ “intermediary capabilities” for effective interaction with AI.

### Constructing an “AI+Peer+Teacher” hybrid integrated feedback

#### Model.

The research findings demonstrate that the AI-assisted peer feedback class achieved higher average scores than the teacher feedback class in the second task, exhibiting a consistent “learning curve effect.” This indicates the significant value of constructing a hybrid integrated feedback model. A feasible system integration approach involves: during the initial drafting phase, AI provides real-time standardized content logic guidance and basic error marking; in the in-depth revision phase, peer collaboration focuses on deep mutual evaluation and negotiation; and in the final polishing phase, teachers offer “expert” high-level feedback targeting areas beyond AI and peer reach—such as optimizing complex syntactic structures and personalized argumentation strategies. This model aims to systematically integrate the “breadth” of tool-mediated support, the “depth” of expert guidance, and the “extent” of social interaction, thereby effectively supporting learners in achieving continuous leaps across different levels of ZPDs.

## Conclusion

The findings in this study indicate distinct yet complementary patterns in the characteristics and effects of teacher feedback and AI-assisted peer feedback as scaffolding mechanisms in writing instruction. Teacher feedback was characterized by comprehensive coverage and dynamic adjustment, with its focus gradually shifting from basic linguistic accuracy to higher-order structural and argumentative development. In contrast, AI-assisted peer feedback demonstrated sustained focus and efficient guidance, consistently emphasizing content quality and logical organization. In terms of score development over time, teacher feedback produced notable short-term gains during the initial intervention stage, largely attributable to its authority and breadth, but this improvement trajectory tended to plateau thereafter. By comparison, AI-assisted peer feedback showed a more stable learning-curve pattern, with its advantages emerging gradually yet persisting over time. With respect to micro-linguistic outcomes, the effects of both scaffolding types appeared bounded and hierarchical: AI-assisted peer feedback facilitated lexical diversity only in the initial task without maintaining this advantage, and neither feedback mode yielded statistically significant improvements in syntactic complexity.

Grounded in Vygotsky’s Sociocultural Theory framework, this study confirms that teacher feedback and AI-assisted peer feedback are “heterogeneous scaffolds” with distinct natures and complementary strengths. Teacher feedback, as an “expert dynamic scaffold,” demonstrates advantages in situational sensitivity and deep regulation. AI-assisted peer feedback, as a “technologically mediated and socially collaborative scaffold,” shows significant efficiency in providing immediate, standardized cognitive support. A single mode cannot comprehensively address the multidimensional challenges of L2 writing ability development. Therefore, constructing a multi-level, dynamically complementary “AI-Peer-Teacher” hybrid integrated feedback model to form a more complete network of mediational activity—supporting learners in achieving continuous leaps across different levels of ZPDs—holds significant theoretical and practical value.

Finally, it is important to note that this study has certain limitations: Firstly, the intervention period was relatively short (only 8 weeks), and the sample consisted solely of English majors from a single university, which restricts the generalizability of the findings. Secondly, the study primarily focused on overall feedback patterns and score changes, lacking in-depth qualitative analysis of how learners engage with and internalize different types of feedback through specific cognitive and social processes, making it difficult to elucidate the specific micro-level mechanisms underlying the two feedback mechanisms. Future research could, on one hand, extend the intervention period and broaden the sample to include learners from diverse academic backgrounds and language proficiency levels, thereby enhancing the universality of the conclusions; on the other hand, develop targeted mediating strategies focusing on specific dimensions of syntactic complexity, and employ qualitative methods such as interviews and audio thinking to thoroughly investigate the cognitive and social processes through which learners internalize different feedback types, thereby providing richer empirical foundations for related studies.

## Supporting information

S1 Table Five-dimensional feedback checklist (Detailed version). (DOCX)
