# Supplementary material for: Teacher feedback VS AI-assisted peer feedback in L2 writing: A quasi-experimental study in a Chinese University
Source: PLoS One. 2026 Jun 29;21(6):e0345976. doi: 10.1371/journal.pone.0345976 (PMC13313374; doi:10.1371/journal.pone.0345976)
Supplement: S1 Table — (DOCX) [file pone.0345976.s001.docx]

**Teacher Feedback VS AI-Assisted Peer Feedback in L2 Writing: A Quasi-Experimental Study in a Chinese University**

**Appendix**

**Five-Dimensional Feedback Checklist (Detailed Version)**

| **Error Category** | **Description** |
| --- | --- |
| **1. Organizational Structure** | The article is **incomplete**, lacking essential components (e.g., title, introduction, conclusion). **Unreasonable structure** with an abnormal number of paragraphs (e.g., only one paragraph or too many). **Disorganized paragraph arrangement**, lacking clear topic sentences, transition phrases, or coherence between paragraphs. **Insufficient argumentation**, lacking supporting evidence such as facts, cases, or data. |
| **2. Content and Theme Expression** | **Unclear viewpoint**, failing to clearly express a personal stance or core argument.  **Unclear expression**, with chaotic sentence structure or **loose logic**.  Prominent use of **run-on sentences** (excessively long, and logically unclear).  Excessive use of **Chinglish expressions**, with sentence structures influenced by **direct translation** from Chinese, resulting in unnatural expression.  Inadequate or improper use of **cohesive devices**. |
| **3. Grammar Errors** | Errors in the use of **noun number** (singular/plural).  Errors in **subject-verb agreement**(disagreement in number, person, or tense).  Errors in **verb tense, voice, or form**.  Missing **sentence elements** or chaotic sentence structure.  Errors in the use of **non-finite verbs** (infinitives, gerunds, participles). |
| **4. Vocabulary (Missing, Misused, or Redundant)** | Missing, misuse, or redundancy of **determiners** (articles, possessive pronouns, demonstrative pronouns, etc.).  Missing, misuse, redundancy of **nouns**, or errors in the possessive case.  Missing, misuse, or redundancy of **adjectives or adverbs** (including errors in the comparative and superlative degrees).  Missing, misuse, or redundancy of **pronouns**.  Missing, misuse, or redundancy of **conjunctions.**  Missing, misuse, or redundancy of **prepositions.**  Missing, misuse, or redundancy of**verbs**.  Errors in the use of **collocations**. |
| **5. Technical Details** | Missing, redundant, or incorrect use of **punctuation**.  Non-standard use of **capitalization.**  **Spelling** errors or **formatting** issues (e.g., typesetting errors like indentation, alignment, line spacing). |
