# Supplementary material for: Teacher feedback VS AI-assisted peer feedback in L2 writing: A quasi-experimental study in a Chinese University
Source: PLoS One. 2026 Jun 29;21(6):e0345976. doi: 10.1371/journal.pone.0345976 (PMC13313374; doi:10.1371/journal.pone.0345976)
Supplement: Attachment — Submitted filename: Response to Reviewers.docx [file pone.0345976.s003.docx]

**Response to Reviewers**

Dear Editor and Reviewers,

Thank you very much for your careful reading and constructive comments on our manuscript (Manuscript ID: [PONE-D-26-08099]). We deeply appreciate the recognition of the timeliness and significance of our research topic, as well as the clarity of our research design and the solidity of our dataset. We have taken all your comments seriously and made substantial revisions accordingly. Below, we address each point in detail.

1. ****Response to** style requirements**（Query #1, by Academic Editor）

Response: Thank you for reminding us of PLOS ONE’s style requirements, including those for file naming. We have carefully reviewed the journal’s submission guidelines and ensured that our manuscript complies fully with the formatting specifications described therein.

2. ****Response to Editorial Queries (Funding Statement & Acknowledgments)****

Response: Thank you for your careful review of our manuscript and for pointing out the inconsistencies regarding funding information. We appreciate your guidance on this matter. We decide to ****remove the Acknowledgments section entirely from the manuscript****. In addition, the projects listed are all completed, so no funding-related text will appear anywhere in the manuscript. We would like to update our ****Funding Statement**** (in the online submission form) to the following:

****“The authors received no specific funding for this work.”****(Query #2, #3, #4 and #5, by Academic Editor).

Please note that this statement is accurate for the current version of the manuscript, as we have chosen not to report the now‑closed grants. We have therefore left the Funding Statement unchanged from the original submission, and we confirm that no funding information is presented elsewhere in the manuscript.

3. Regarding the **recommendation to review and evaluate specific previously published works** mentioned in the reviewer comments（Query #6, by Academic Editor）, we have carefully assessed each suggested reference against the focus and scope of our study. We confirm that the references currently cited in our manuscript are all closely aligned with the research content and theoretical framework.

In this revision, to enhance conciseness and sharpen the scholarly focus of the manuscript, we have streamlined the reference list by removing several tangentially related citations. At the same time, we have added a small number of highly relevant publications that strengthen the theoretical grounding and empirical contextualization of our study. The updated reference list now reflects a more precise and purposeful selection of literature that directly supports our arguments.

We trust that these revisions meet the journal’s requirements.

**Response to Reviewer** #**1：**

**Comment 1:** In the abstract, it is recommended to present the research gap after outlining the research background and then introduce the research content. Otherwise, the study’s innovation is difficult to highlight. In addition, this section does not reflect the contributions of the study.
Response: Thank you greatly for this valuable and constructive comment. We fully agree that the original abstract failed to clearly present the research gaps and adequately highlight the theoretical innovations and practical contributions of this study, which affected the logical rigor and readability of the manuscript.

In accordance with your suggestion, we have thoroughly restructured and revised the abstract. First, we briefly outlined the research background concerning the application of AI-assisted feedback in L2 writing instruction. Second, we explicitly clarified the existing research gaps, namely, previous literature lacks systematic sociocultural comparisons between teacher feedback and AI-assisted peer feedback, and neglects their dynamic evolutionary features, micro-linguistic effects, and integrated feedback frameworks. Afterwards, we clearly introduced the research design, methodological procedures, and core empirical findings. Finally, we fully supplemented and highlighted the theoretical innovations and practical contributions of this study.

The revised abstract logically progresses from research background → research gaps → research design → key findings → theoretical and practical contributions, which effectively highlights the originality and significance of the present study. Please refer to the revised manuscript for details.

**Comment 2:** In the introduction, there is a logical jump between the first two sentences of the opening paragraph. In addition, the statement of the research gap in this section is not sufficiently clear. Moreover, the connection between the theoretical foundation and the present study is not close enough.

Response: Thank you for the feedback. We revised the opening sentence to clarify context and logical flow. The research gap and theoretical connection will be further strengthened in the revised manuscript.

Original Text: Writing, as a core product of Second Language Acquisition (SLA), directly reflects the internalization degree of a learner’s second language proficiency. According to Lee (2017)[1], feedback is a mechanism that promotes learning by providing valuable information about an individual’s performance. Early research primarily focused on the nature and efficacy of feedback from teachers, peers, and platforms like Criterion. However, with the rise of generative AI tools like ChatGPT, AI-assisted feedback has become a new mediator in L2 writing instruction due to its advantages in content guidance and deep logical analysis[2,3,4,5].

Revised Text: Writing, as a core product of Second Language Acquisition (SLA), directly reflects the internalization degree of a learner’s second language proficiency. In the context of L2 writing, feedback serves as a crucial link between teaching and learning, facilitating learning by providing valuable insights into individual performance[1]. Over the past two decades, research on L2 writing feedback has evolved from descriptive accounts to empirical investigations and shifted from single-method designs to more diversified and integrated research approaches. Early studies predominantly focused on the effectiveness of feedback delivered by teachers, peers, and automated writing evaluation (AWE) platforms such as Criterion. With the emergence of generative AI tools represented by ChatGPT in recent years, AI-based feedback has become an innovative mediating tool in L2 writing instruction due to its strengths in content guidance and in-depth logical analysis.

**Comment 3:** In the literature review, it is recommended to place the review of the theoretical foundation at the beginning. In addition, this section lacks definitions of the core concepts. Overall, the organization of the literature review is rather loose, and the discussions of AI feedback and teacher feedback in different parts could be further integrated. Moreover, the statement of the research gap in this section remains relatively vague.

Response: Thank you for the detailed suggestions. We have restructured the literature review by placing the theoretical foundation first, clearly define core concepts, better integrate discussions of AI and teacher feedback, and sharpen the research gap statement.

**Comment 4:** Regarding the participants and research design, it is recommended to clarify the basis for group assignment, such as whether the groups were formed according to intact classes or through random assignment. In addition, this section lacks a description of the sample background, including the gender distribution, English proficiency, prior writing training, and experience with AI tools.

Response: Thank you for the suggestion. We have clarified that the two groups were formed from two intact natural classes, with baseline writing proficiency showing no significant difference. We have also added the missing sample background information (gender distribution, prior writing training, and prior AI experience) in the revised manuscript.

**Comment 5:** Regarding the research instruments, it is recommended to provide further information on the basis for adapting the standardized five-dimensional feedback checklist, as well as whether the adaptation was reviewed by experts and supported by evidence of validity or appropriateness.

Response: Regarding your review comments on “the rationale for revising the standardized five-dimensional feedback checklist, expert review, and evidence supporting its validity,” we have made corresponding improvements in the revised draft.

**Comment 6:** In the discussion, you state that this study integrates quantitative and qualitative data. However, neither the results section nor the discussion section presents any qualitative data.

Response: We highly appreciate this valuable comment. Although qualitative data were collected through interviews in the preliminary stage of this study, the data analysis and result discussion of this research are mainly based on quantitative data, and the qualitative data were not adopted as the core analytical materials in the formal manuscript. To ensure complete consistency among the research design, analytical process and textual statements, and eliminate potential ambiguity, we have removed all descriptions related to qualitative research throughout the manuscript. All relevant expressions have been uniformly revised to indicate that data analysis and result discussion are conducted based on quantitative data, thereby ensuring the rigor and logical consistency of the manuscript.

**Comment 7:** In the discussion section, the study does not engage sufficiently with previous studies. To make this section more substantial, you may cite the following studies:

1. Derakhshan, A., & Park., Y. (2026a). The role of multimodal AI technologies in EFL students’ perceived positive and negative achievement emotions: An existential positive psychology (EPP) perspective. Language Related Research, 17(3), 1–27. [https://doi.org/10.48311/lrr.2025.118514.83043](https://doi.org/10.48311/lrr.2025.118514.83043)
2. Derakhshan, A., & Park, Y. (2026b). Exploring the role of AI adoption in under-resourced students' psychological needs satisfaction and frustration: A fresh perspective from METUX. Journal of Education for Students Placed at Risk (JESPAR). [https://doi.org/10.1080/10824669.2026.2625660](https://doi.org/10.1080/10824669.2026.2625660)
3. Yang, L., & Derakhshan, A. (2026). Unpacking the factors shaping TESOL teachers’ GenAI literacy from an ecological perspective. TESOL Quarterly, 1-26. [https://doi.org/10.1002/tesq.70095](https://doi.org/10.1002/tesq.70095)
4. Yang, L. (2026). Empowering the autonomous learner: How AI-assisted language learning environments shape self-regulation, autonomy, and self-directed behaviors. Language Teaching Research, 0(0), 1-23. [https://doi.org/10.1177/13621688261422129](https://doi.org/10.1177/13621688261422129)
5. Yao, N., Yang, L., & Mutlu, A. K. (2025). Teacher interpersonal behaviours, communication apprehension, self-efficacy and academic engagement in technology-mediated EFL classrooms: A cross-sectional study of Mongolian trilingual learners. European Journal of Education, 61(1), 1-13. [https://doi.org/10.1111/ejed.70397](https://doi.org/10.1111/ejed.70397)

Response: Thank you for your valuable suggestion and for recommending the relevant references. We have carefully read the five papers you suggested and have cited three of them (Derakhshan & Park, 2026a; Derakhshan & Park, 2026b; Yang, 2026) in the revised paper, as they are highly relevant to our discussions on AI‑assisted language learning, learner emotions, and student engagement, thereby enhancing the engagement with current literature. The other two studies, while academically valuable, focus primarily on teachers’ GenAI literacy, as well as Teacher interpersonal behaviours, communication apprehension, self-efficacy and academic engagement , which differ from the specific focus of the present study on feedback mechanisms and writing development pathways. Therefore, they were not cited in the manuscript. We appreciate your thoughtful guidance.

**Comment 8：**In the conclusion, the limitations are not well aligned with the directions for future research.

Response：We carefully reviewed the statements regarding limitations and future research directions in the conclusion section and found that the original text indeed failed to adequately address the second limitation (namely, the lack of a deep qualitative analysis of the learner's internalization of feedback). Consequently, we have made targeted revisions. The revised version retains the original focus on extending the study duration, expanding the sample size, and designing syntactic strategies, while also incorporating qualitative research methods, thereby directly addressing the limitation of “lack of deep qualitative analysis.” The current limitations and future research directions are now well aligned.

**Response to Reviewer** #**2：**

**Reviewer #2: 1.** The topic is highly relevant, addressing a critical gap in L2 writing research: the comparative efficacy of traditional teacher feedback and emerging AI-mediated collaborative feedback. The practical implications are clear, especially for application-oriented institutions seeking efficient, scalable feedback models. The theoretical framing within Sociocultural Theory is appropriate, though the potential to contribute to broader debates in SLA (e.g., about mediation, internalization, and the role of technology in scaffolding) could be more explicitly articulated.

Response：We extend our sincere gratitude for your recognition of the relevance of this research topic and the applicability of its theoretical framework. We particularly appreciate your valuable suggestion to clearly articulate the “contribution to broader debates in SLA”. Based on your feedback, we have strategically enhanced the discussion of the broader SLA issues in the introduction and literature review sections of the revised draft, particularly in the theoretical framework and the identification of research gaps. Key revisions include: Regarding mediation and internalization mechanisms, we explicitly state in “Literature Review——Theoretical Framework: Sociocultural Theory and Feedback in Second Language Writing” that “feedback essentially serves as an external scaffold whose value depends on its precise alignment with the learner’s cognitive potential within the ZPD and its gradual withdrawal at appropriate moments, thereby enabling learners to develop independent task completion skills.” We also emphasize the “dialogic” and “contextualized” nature of written feedback, thereby directly incorporating the mediation and internalization processes into our analytical framework.
Regarding the role of technology in scaffolding: In “Literature Review”, we specifically examined the theoretical limitations of the traditional SCT framework, which categorizes technological tools merely as static auxiliary intermediaries, and noted that “with the emergence of generative AI, the scaffolding attribute of AI remains inadequately theorized.” We explicitly defined AI-assisted peer feedback as a “social-technical composite scaffold.” This discussion directly addresses your inquiry into “the role of technology in scaffolding-based teaching.”
In the section on research gaps and contributions, we explicitly state that this study aims to conduct a systematic comparative analysis of the scaffolding effects of teacher feedback versus AI-assisted peer feedback within the SCT framework, thereby providing empirical evidence for the effectiveness of ‘technology-mediated social collaboration scaffolding.’ This approach closely aligns the research contributions with the core theoretical debates in SLA—mediation, internalization, and technological scaffolding.
The aforementioned revisions have provided a clearer and more in-depth elaboration of this study’s potential contribution to the broader issues of SLA. Once again, we extend our gratitude for your meticulous guidance and constructive feedback.

**Reviewer #2: 2.** The literature review is comprehensive, covering key areas: teacher feedback, peer feedback, AWE, and generative AI feedback. It successfully identifies a research gap—the lack of comparative studies within a unified theoretical framework—and positions the study accordingly. However, the review tends toward description rather than critical synthesis. For instance, while AI-assisted peer feedback is presented as a novel “technologically-intermediary social collaborative scaffold,” the theoretical distinctions between this and other mediated feedback forms (e.g., teacher-guided peer feedback) are not sufficiently problematized. A sharper critique of existing conceptual binaries (expert vs. tool) would strengthen the rationale. The integration of theory into the analysis could be deeper. For example, the discussion of “scaffolding” often remains metaphorical; a more detailed analysis of how each feedback type operates as a mediational tool within the students’ ZPD—perhaps linked to specific interactional data from the peer negotiations—would enhance the theoretical contribution.

Response：In response to your specific concerns, we have made the following adjustments: 1. We enhanced critical synthesis rather than mere description: By reconstructing “Theoretical Framework: Sociocultural Theory and Feedback in Second Language Writing” and “From ‘Substitution’ to ‘Collaboration’: A Paradigm Shift in Integration Approaches,” we elevated the metaphor beyond purely descriptive narratives. 2. Deepening the theoretical analysis of “scaffolding” and ZPD: We fully agree that “scaffolding” should not remain merely metaphorical. We expanded our analysis of how each feedback type operates within the ZPD framework. We clarified that teacher feedback relies on “dynamic adaptation to the individual’s ZPD,” whereas AI-PF provides “standardized cognitive support” to ensure comprehensive coverage. We believe these modifications significantly enhance the theoretical depth of the paper and clearly define the study's position within the SLA discourse framework.

**Reviewer #2: 3.** One concern is the handling of the linguistic complexity data: the non-significant findings for syntactic complexity are acknowledged but not fully theorized within the SCT framework. Are these aspects beyond the reach of these scaffolds, or was the intervention duration insufficient? A more nuanced interpretation is needed.

Response：Following your recommendations, we have conducted a more detailed theoretical analysis of the non-significant results regarding syntactic complexity in the discussion section of the revised draft, with specific additions as follows: Syntactic complexity is explicitly categorized under the “high-level ZPD” framework; we further emphasize that the non-significant findings regarding syntactic complexity in this study are more likely attributable to mismatches between scaffold types and language development stages, rather than simply insufficient intervention duration or exceeding the scope of existing theoretical frameworks. Specifically: While teacher feedback exhibits dynamic adaptability, its focus naturally shifts from basic linguistic forms to content and structure within limited class hours, making sustained emphasis on syntactic dimensions challenging; although AI-assisted peer feedback provides immediate suggestions, its general language models have not yet systematically identified learners' syntactic weaknesses for targeted training. Therefore, enhancing syntactic complexity may require an intervention period longer than the 8 weeks in this study, along with specialized mediating strategies tailored to syntactic development rather than relying on general writing feedback.

**Reviewer #2: 4.** The empirical data are substantial (244 writing samples, 61 students), and the collection procedures are clearly described. The use of established instruments (e.g., Coh-Metrix indices) and reporting of reliability metrics (e.g., Cronbach’s α for questionnaires) are commendable. However, the qualitative component is underdeveloped. The manuscript mentions semi-structured interviews but provides no excerpts, thematic analysis, or discussion of how this data informed the findings. Integrating qualitative insights (e.g., on student perception of scaffold types) would greatly enrich the discussion. References are comprehensive and relevant.

Response：We highly appreciate this valuable comment. As previously mentioned, qualitative data were collected through interviews in the preliminary stage of this study, the data analysis and result discussion of this research are mainly based on quantitative data, and the qualitative data were not adopted as the core analytical materials in the formal manuscript. To ensure complete consistency among the research design, analytical process and textual statements, and eliminate potential ambiguity, we have removed all descriptions related to qualitative research throughout the manuscript. All relevant expressions have been uniformly revised to indicate that data analysis and result discussion are conducted based on quantitative data, thereby ensuring the rigor and logical consistency of the manuscript.

**Reviewer #2: 5.** Discuss how the 8-week duration, the specific AI tool (DeepSeek), and the task type (argumentative essays) might limit generalizability. Suggest how future studies could overcome these.

Response：We fully endorse this perspective and have specifically supplemented and discussed it in the conclusion section (limitation section) of the revised draft. The specific revisions are as follows: Regarding the intervention duration (8 weeks): We explicitly stated in the limitations section that the shorter intervention period limits the ability to observe long-term scaffold effects and the language internalization process. Therefore, for future research directions, we recommend extending the intervention duration to one semester or longer and employing a longitudinal tracking design to examine the sustained impact of the two feedback modes on writing ability development and the persistence of effects after scaffold removal.

Regarding the specific AI tool: We note that different generative AI tools differ in feedback accuracy, interaction style, and task adaptability. The results obtained from DeepSeek alone in this study should not be directly generalized to all AI tools. Future research should compare the similarities and differences among various AI tools in assisting peer feedback and examine the mediating role of tool characteristics (e.g., response speed, explanatory depth, and multi-turn dialogue capabilities) on feedback effectiveness.

Regarding task types (argumentative essays): We acknowledge that argumentative writing emphasizes logical reasoning and structural organization, whereas other genres (such as narrative, expository, and practical writing) may have distinct requirements for linguistic form, creative expression, or normative standards. The findings of this study may not apply universally to all writing tasks. Future research may encompass multiple writing genres to examine the consistency of the two feedback modes across different task types and to determine whether feedback scaffolds need to be tailored to genre-specific characteristics.

We once again appreciate your meticulous feedback, as these suggestions have significantly enhanced our clear understanding of the applicable boundaries of the study’s findings. Thank you all for your suggestions and guidance.
