# Supplementary material for: Teacher feedback VS AI-assisted peer feedback in L2 writing: A quasi-experimental study in a Chinese University
Source: PLoS One. 2026 Jun 29;21(6):e0345976. doi: 10.1371/journal.pone.0345976 (PMC13313374; doi:10.1371/journal.pone.0345976)
Supplement: Peer review history

# Peer review: Decision Letter 0

**DOI:** 10.1371/journal.pone.0345976.r001

**Authors:** Ali Derakhshan

13 Apr 2026

PONE-D-26-08099

Teacher Feedback VS AI-Assisted Peer Feedback in L2 Writing: A Quasi-Experimental Study in a Chinese University

PLOS One

Dear Dr. Luo,

Thank you for submitting your manuscript to PLOS ONE. After careful consideration, we feel that it has merit but does not fully meet PLOS ONE’s publication criteria as it currently stands. Therefore, we invite you to submit a revised version of the manuscript that addresses the points raised during the review process.

==============================

**ACADEMIC EDITOR:**

Dear Rong,

Thank you for your submission. Please revise your paper, highlight the changes, and provide a response letter. You need to improve your review of literature, strengthen your discussion by linking it to the conceptual framework, and expand the implications.

Best,

Ali Derakhshan

==============================

Please submit your revised manuscript by May 28 2026 11:59PM. If you will need more time than this to complete your revisions, please reply to this message or contact the journal office at plosone@plos.org. When you're ready to submit your revision, log on to https://www.editorialmanager.com/pone/ and select the 'Submissions Needing Revision' folder to locate your manuscript file.

Please include the following items when submitting your revised manuscript:

- A letter that responds to each point raised by the academic editor and reviewer(s). You should upload this letter as a separate file labeled 'Response to Reviewers'.
- A marked-up copy of your manuscript that highlights changes made to the original version. You should upload this as a separate file labeled 'Revised Manuscript with Track Changes'.
- An unmarked version of your revised paper without tracked changes. You should upload this as a separate file labeled 'Manuscript'.

If you would like to make changes to your financial disclosure, please include your updated statement in your cover letter. Guidelines for resubmitting your figure files are available below the reviewer comments at the end of this letter.

If applicable, we recommend that you deposit your laboratory protocols in protocols.io to enhance the reproducibility of your results. Protocols.io assigns your protocol its own identifier (DOI) so that it can be cited independently in the future. For instructions see: https://journals.plos.org/plosone/s/submission-guidelines#loc-laboratory-protocols. Additionally, PLOS ONE offers an option for publishing peer-reviewed Lab Protocol articles, which describe protocols hosted on protocols.io. Read more information on sharing protocols at https://plos.org/protocols?utm_medium=editorial-email&utm_source=authorletters&utm_campaign=protocols.

As the corresponding author, your ORCID iD is verified in the submission system and will appear in the published article. PLOS supports the use of ORCID, and we encourage all coauthors to register for an ORCID iD and use it as well. Please encourage your coauthors to verify their ORCID iD within the submission system before final acceptance, as unverified ORCID iDs will not appear in the published article. *Only*  the individual author can complete the verification step; PLOS staff *cannot*  verify ORCID iDs on behalf of authors.

We look forward to receiving your revised manuscript.

Kind regards,

Ali Derakhshan

Academic Editor

PLOS One

Journal Requirements:

When submitting your revision, we need you to address these additional requirements.

1. Please ensure that your manuscript meets PLOS ONE's style requirements, including those for file naming. The PLOS ONE style templates can be found at

https://journals.plos.org/plosone/s/file?id=wjVg/PLOSOne_formatting_sample_main_body.pdf and

https://journals.plos.org/plosone/s/file?id=ba62/PLOSOne_formatting_sample_title_authors_affiliations.pdf

2. Thank you for stating the following in the Acknowledgments Section of your manuscript:

"This research was supported by Education Research Project of Wuhan Municipal Department of Education (Grant No. 2021083) , Ministry of Education Industry- Academia Collaboration Projects (Grant No. 20241058), and Research on Digital Transformation of Higher Education in Colleges and Universities for the Year 2025 by the Association for Employment of College Graduates(Grant No. GJX25Z2144). The sponsors were not involved in designing the study, gathering and analyzing data, deciding to publish, or drafting the manuscript."

We note that you have provided additional information within the Acknowledgements Section that is not currently declared in your Funding Statement. Please note that funding information should not appear in the Acknowledgments section or other areas of your manuscript. We will only publish funding information present in the Funding Statement section of the online submission form.

Please remove any funding-related text from the manuscript and let us know how you would like to update your Funding Statement. Currently, your Funding Statement reads as follows:

"The author(s) received no specific funding for this work."

Please include your amended statements within your cover letter; we will change the online submission form on your behalf.

3. We note that the grant information you provided in the ‘Funding Information’ and ‘Financial Disclosure’ sections do not match.

When you resubmit, please ensure that you provide the correct grant numbers for the awards you received for your study in the ‘Funding Information’ section.

4. Thank you for stating the following financial disclosure:

"The author(s) received no specific funding for this work."

At this time, please address the following queries:

a)        Please clarify the sources of funding (financial or material support) for your study. List the grants or organizations that supported your study, including funding received from your institution.

b)        State what role the funders took in the study. If the funders had no role in your study, please state: “The funders had no role in study design, data collection and analysis, decision to publish, or preparation of the manuscript.”

c)        If any authors received a salary from any of your funders, please state which authors and which funders.

d)        If you did not receive any funding for this study, please state: “The authors received no specific funding for this work.”

Please include your amended statements within your cover letter; we will change the online submission form on your behalf.

5. Please include captions for your Supporting Information files at the end of your manuscript, and update any in-text citations to match accordingly. Please see our Supporting Information guidelines for more information: http://journals.plos.org/plosone/s/supporting-information.

6. If the reviewer comments include a recommendation to cite specific previously published works, please review and evaluate these publications to determine whether they are relevant and should be cited. There is no requirement to cite these works unless the editor has indicated otherwise.

Additional Editor Comments:

Dear Rong,

Thank you for your submission. Please revise your paper, highlight the changes, and provide a response letter. You need to improve your review of literature, strengthen your discussion by linking it to the conceptual framework, and expand the implications.

Best,

Ali Derakhshan

[Note: HTML markup is below. Please do not edit.]

Reviewers' comments:

Reviewer's Responses to Questions

**Comments to the Author**

1. Is the manuscript technically sound, and do the data support the conclusions?

The manuscript must describe a technically sound piece of scientific research with data that supports the conclusions. Experiments must have been conducted rigorously, with appropriate controls, replication, and sample sizes. The conclusions must be drawn appropriately based on the data presented.

Reviewer #1: Yes

Reviewer #2: Yes

**********

2. Has the statistical analysis been performed appropriately and rigorously?

Reviewer #1: Yes

Reviewer #2: Yes

**********

3. Have the authors made all data underlying the findings in their manuscript fully available?

The PLOS Data policy requires authors to make all data underlying the findings described in their manuscript fully available without restriction, with rare exception (please refer to the Data Availability Statement in the manuscript PDF file). The data should be provided as part of the manuscript or its supporting information, or deposited to a public repository. For example, in addition to summary statistics, the data points behind means, medians and variance measures should be available. If there are restrictions on publicly sharing data—e.g. participant privacy or use of data from a third party—those must be specified.

Reviewer #1: Yes

Reviewer #2: Yes

**********

4. Is the manuscript presented in an intelligible fashion and written in standard English?

PLOS ONE does not copyedit accepted manuscripts, so the language in submitted articles must be clear, correct, and unambiguous. Any typographical or grammatical errors should be corrected at revision, so please note any specific errors here.

Reviewer #1: Yes

Reviewer #2: Yes

**********

5. Review Comments to the Author

Please use the space provided to explain your answers to the questions above. You may also include additional comments for the author, including concerns about dual publication, research ethics, or publication ethics. (Please upload your review as an attachment if it exceeds 20,000 characters)

Reviewer #1: Dear author(s):

I have thoroughly reviewed your study and appreciate the effort that you put into it. However, I would like to address several aspects that could further enhance the quality of your work. Please consider the following comments to refine your study.

In the abstract, it is recommended to present the research gap after outlining the research background and then introduce the research content. Otherwise, the study’s innovation is difficult to highlight. In addition, this section does not reflect the contributions of the study.

In the introduction, there is a logical jump between the first two sentences of the opening paragraph. In addition, the statement of the research gap in this section is not sufficiently clear. Moreover, the connection between the theoretical foundation and the present study is not close enough.

In the literature review, it is recommended to place the review of the theoretical foundation at the beginning. In addition, this section lacks definitions of the core concepts. Overall, the organization of the literature review is rather loose, and the discussions of AI feedback and teacher feedback in different parts could be further integrated. Moreover, the statement of the research gap in this section remains relatively vague.

Regarding the participants and research design, it is recommended to clarify the basis for group assignment, such as whether the groups were formed according to intact classes or through random assignment. In addition, this section lacks a description of the sample background, including the gender distribution, English proficiency, prior writing training, and experience with AI tools.

Regarding the research instruments, it is recommended to provide further information on the basis for adapting the standardized five-dimensional feedback checklist, as well as whether the adaptation was reviewed by experts and supported by evidence of validity or appropriateness.

In the discussion, you state that this study integrates quantitative and qualitative data. However, neither the results section nor the discussion section presents any qualitative data.

In the discussion section, the study does not engage sufficiently with previous studies. To make this section more substantial, you may cite the following studies:

Derakhshan, A., & Park., Y. (2026b). The role of multimodal AI technologies in EFL students’ perceived positive and negative achievement emotions: An existential positive psychology (EPP) perspective. Language Related Research, 17(3), 1–27. https://doi.org/10.48311/lrr.2025.118514.83043

Derakhshan, A., & Park, Y. (2026a). Exploring the role of AI adoption in under-resourced students' psychological needs satisfaction and frustration: A fresh perspective from METUX. Journal of Education for Students Placed at Risk (JESPAR). https://doi.org/10.1080/10824669.2026.2625660

Yang, L., & Derakhshan, A. (2026). Unpacking the factors shaping TESOL teachers’ GenAI literacy from an ecological perspective. TESOL Quarterly, 1-26. https://doi.org/10.1002/tesq.70095

Yang, L. (2026). Empowering the autonomous learner: How AI-assisted language learning environments shape self-regulation, autonomy, and self-directed behaviors. Language Teaching Research, 0(0), 1-23. https://doi.org/10.1177/13621688261422129

Yao, N., Yang, L., & Mutlu, A. K. (2025). Teacher interpersonal behaviours, communication apprehension, self-efficacy and academic engagement in technology-mediated EFL classrooms: A cross-sectional study of Mongolian trilingual learners. European Journal of Education, 61(1), 1-13. https://doi.org/10.1111/ejed.70397

In the conclusion, the limitations are not well aligned with the directions for future research.

Thank you for considering my review comments, and I hope these suggestions will be helpful in further improving your manuscript.

Sincerely,

Reviewer #2: 1. The topic is highly relevant, addressing a critical gap in L2 writing research: the comparative efficacy of traditional teacher feedback and emerging AI-mediated collaborative feedback. The practical implications are clear, especially for application-oriented institutions seeking efficient, scalable feedback models. The theoretical framing within Sociocultural Theory is appropriate, though the potential to contribute to broader debates in SLA (e.g., about mediation, internalization, and the role of technology in scaffolding) could be more explicitly articulated.

2. The literature review is comprehensive, covering key areas: teacher feedback, peer feedback, AWE, and generative AI feedback. It successfully identifies a research gap—the lack of comparative studies within a unified theoretical framework—and positions the study accordingly. However, the review tends toward description rather than critical synthesis. For instance, while AI-assisted peer feedback is presented as a novel “technologically-intermediary social collaborative scaffold,” the theoretical distinctions between this and other mediated feedback forms (e.g., teacher-guided peer feedback) are not sufficiently problematized. A sharper critique of existing conceptual binaries (expert vs. tool) would strengthen the rationale. The integration of theory into the analysis could be deeper. For example, the discussion of “scaffolding” often remains metaphorical; a more detailed analysis of how each feedback type operates as a mediational tool within the students’ ZPD—perhaps linked to specific interactional data from the peer negotiations—would enhance the theoretical contribution.

3. One concern is the handling of the linguistic complexity data: the non-significant findings for syntactic complexity are acknowledged but not fully theorized within the SCT framework. Are these aspects beyond the reach of these scaffolds, or was the intervention duration insufficient? A more nuanced interpretation is needed.

4. The empirical data are substantial (244 writing samples, 61 students), and the collection procedures are clearly described. The use of established instruments (e.g., Coh-Metrix indices) and reporting of reliability metrics (e.g., Cronbach’s α for questionnaires) are commendable. However, the qualitative component is underdeveloped. The manuscript mentions semi-structured interviews but provides no excerpts, thematic analysis, or discussion of how this data informed the findings. Integrating qualitative insights (e.g., on student perception of scaffold types) would greatly enrich the discussion. References are comprehensive and relevant.

5. Discuss how the 8-week duration, the specific AI tool (DeepSeek), and the task type (argumentative essays) might limit generalizability. Suggest how future studies could overcome these.

**********

6. PLOS authors have the option to publish the peer review history of their article (what does this mean?). If published, this will include your full peer review and any attached files.

If you choose “no”, your identity will remain anonymous but your review may still be made public.

**Do you want your identity to be public for this peer review?**  For information about this choice, including consent withdrawal, please see our Privacy Policy.

Reviewer #1: No

Reviewer #2: No

**********

[NOTE: If reviewer comments were submitted as an attachment file, they will be attached to this email and accessible via the submission site. Please log into your account, locate the manuscript record, and check for the action link "View Attachments". If this link does not appear, there are no attachment files.]

To ensure your figures meet our technical requirements, please review our figure guidelines: https://journals.plos.org/plosone/s/figures

You may also use PLOS’s free figure tool, NAAS, to help you prepare publication quality figures: https://journals.plos.org/plosone/s/figures#loc-tools-for-figure-preparation.

NAAS will assess whether your figures meet our technical requirements by comparing each figure against our figure specifications.

# Author comment: Author response to Decision Letter 1

**DOI:** 10.1371/journal.pone.0345976.r002

11 May 2026

Response to Reviewers

Dear Editor and Reviewers,

Thank you very much for your careful reading and constructive comments on our manuscript (Manuscript ID: [PONE-D-26-08099]). We deeply appreciate the recognition of the timeliness and significance of our research topic, as well as the clarity of our research design and the solidity of our dataset. We have taken all your comments seriously and made substantial revisions accordingly. Below, we address each point in detail.

1.Response to style requirements（Query #1, by Academic Editor）

Response: Thank you for reminding us of PLOS ONE’s style requirements, including those for file naming. We have carefully reviewed the journal’s submission guidelines and ensured that our manuscript complies fully with the formatting specifications described therein.

2.Response to Editorial Queries (Funding Statement & Acknowledgments)

Response: Thank you for your careful review of our manuscript and for pointing out the inconsistencies regarding funding information. We appreciate your guidance on this matter. We decide to remove the Acknowledgments section entirely from the manuscript. In addition, the projects listed are all completed, so no funding-related text will appear anywhere in the manuscript. We would like to update our Funding Statement (in the online submission form) to the following:

“The authors received no specific funding for this work.”(Query #2, #3, #4 and #5, by Academic Editor).

Please note that this statement is accurate for the current version of the manuscript, as we have chosen not to report the now closed grants. We have therefore left the Funding Statement unchanged from the original submission, and we confirm that no funding information is presented elsewhere in the manuscript.

3.Regarding the recommendation to review and evaluate specific previously published works mentioned in the reviewer comments（Query #6, by Academic Editor）, we have carefully assessed each suggested reference against the focus and scope of our study. We confirm that the references currently cited in our manuscript are all closely aligned with the research content and theoretical framework.

In this revision, to enhance conciseness and sharpen the scholarly focus of the manuscript, we have streamlined the reference list by removing several tangentially related citations. At the same time, we have added a small number of highly relevant publications that strengthen the theoretical grounding and empirical contextualization of our study. The updated reference list now reflects a more precise and purposeful selection of literature that directly supports our arguments.

We trust that these revisions meet the journal’s requirements.

Response to Reviewer #1：

Comment 1: In the abstract, it is recommended to present the research gap after outlining the research background and then introduce the research content. Otherwise, the study’s innovation is difficult to highlight. In addition, this section does not reflect the contributions of the study.

Response: Thank you greatly for this valuable and constructive comment. We fully agree that the original abstract failed to clearly present the research gaps and adequately highlight the theoretical innovations and practical contributions of this study, which affected the logical rigor and readability of the manuscript.

In accordance with your suggestion, we have thoroughly restructured and revised the abstract. First, we briefly outlined the research background concerning the application of AI-assisted feedback in L2 writing instruction. Second, we explicitly clarified the existing research gaps, namely, previous literature lacks systematic sociocultural comparisons between teacher feedback and AI-assisted peer feedback, and neglects their dynamic evolutionary features, micro-linguistic effects, and integrated feedback frameworks. Afterwards, we clearly introduced the research design, methodological procedures, and core empirical findings. Finally, we fully supplemented and highlighted the theoretical innovations and practical contributions of this study.

The revised abstract logically progresses from research background → research gaps → research design → key findings → theoretical and practical contributions, which effectively highlights the originality and significance of the present study. Please refer to the revised manuscript for details.

Comment 2: In the introduction, there is a logical jump between the first two sentences of the opening paragraph. In addition, the statement of the research gap in this section is not sufficiently clear. Moreover, the connection between the theoretical foundation and the present study is not close enough.

Response: Thank you for the feedback. We revised the opening sentence to clarify context and logical flow. The research gap and theoretical connection will be further strengthened in the revised manuscript.

Original Text: Writing, as a core product of Second Language Acquisition (SLA), directly reflects the internalization degree of a learner’s second language proficiency. According to Lee (2017)[1], feedback is a mechanism that promotes learning by providing valuable information about an individual’s performance. Early research primarily focused on the nature and efficacy of feedback from teachers, peers, and platforms like Criterion. However, with the rise of generative AI tools like ChatGPT, AI-assisted feedback has become a new mediator in L2 writing instruction due to its advantages in content guidance and deep logical analysis[2,3,4,5].

Revised Text: Writing, as a core product of Second Language Acquisition (SLA), directly reflects the internalization degree of a learner’s second language proficiency. In the context of L2 writing, feedback serves as a crucial link between teaching and learning, facilitating learning by providing valuable insights into individual performance[1]. Over the past two decades, research on L2 writing feedback has evolved from descriptive accounts to empirical investigations and shifted from single-method designs to more diversified and integrated research approaches. Early studies predominantly focused on the effectiveness of feedback delivered by teachers, peers, and automated writing evaluation (AWE) platforms such as Criterion. With the emergence of generative AI tools represented by ChatGPT in recent years, AI-based feedback has become an innovative mediating tool in L2 writing instruction due to its strengths in content guidance and in-depth logical analysis.

Comment 3: In the literature review, it is recommended to place the review of the theoretical foundation at the beginning. In addition, this section lacks definitions of the core concepts. Overall, the organization of the literature review is rather loose, and the discussions of AI feedback and teacher feedback in different parts could be further integrated. Moreover, the statement of the research gap in this section remains relatively vague.

Response: Thank you for the detailed suggestions. We have restructured the literature review by placing the theoretical foundation first, clearly define core concepts, better integrate discussions of AI and teacher feedback, and sharpen the research gap statement.

Comment 4: Regarding the participants and research design, it is recommended to clarify the basis for group assignment, such as whether the groups were formed according to intact classes or through random assignment. In addition, this section lacks a description of the sample background, including the gender distribution, English proficiency, prior writing training, and experience with AI tools.

Response: Thank you for the suggestion. We have clarified that the two groups were formed from two intact natural classes, with baseline writing proficiency showing no significant difference. We have also added the missing sample background information (gender distribution, prior writing training, and prior AI experience) in the revised manuscript.

Comment 5: Regarding the research instruments, it is recommended to provide further information on the basis for adapting the standardized five-dimensional feedback checklist, as well as whether the adaptation was reviewed by experts and supported by evidence of validity or appropriateness.

Response: Regarding your review comments on “the rationale for revising the standardized five-dimensional feedback checklist, expert review, and evidence supporting its validity,” we have made corresponding improvements in the revised draft.

Comment 6: In the discussion, you state that this study integrates quantitative and qualitative data. However, neither the results section nor the discussion section presents any qualitative data.

Response: We highly appreciate this valuable comment. Although qualitative data were collected through interviews in the preliminary stage of this study, the data analysis and result discussion of this research are mainly based on quantitative data, and the qualitative data were not adopted as the core analytical materials in the formal manuscript. To ensure complete consistency among the research design, analytical process and textual statements, and eliminate potential ambiguity, we have removed all descriptions related to qualitative research throughout the manuscript. All relevant expressions have been uniformly revised to indicate that data analysis and result discussion are conducted based on quantitative data, thereby ensuring the rigor and logical consistency of the manuscript.

Comment 7: In the discussion section, the study does not engage sufficiently with previous studies. To make this section more substantial, you may cite the following studies:

1.Derakhshan, A., & Park., Y. (2026a). The role of multimodal AI technologies in EFL students’ perceived positive and negative achievement emotions: An existential positive psychology (EPP) perspective. Language Related Research, 17(3), 1–27. https://doi.org/10.48311/lrr.2025.118514.83043

2.Derakhshan, A., & Park, Y. (2026b). Exploring the role of AI adoption in under-resourced students' psychological needs satisfaction and frustration: A fresh perspective from METUX. Journal of Education for Students Placed at Risk (JESPAR). https://doi.org/10.1080/10824669.2026.2625660

3.Yang, L., & Derakhshan, A. (2026). Unpacking the factors shaping TESOL teachers’ GenAI literacy from an ecological perspective. TESOL Quarterly, 1-26. https://doi.org/10.1002/tesq.70095

4.Yang, L. (2026). Empowering the autonomous learner: How AI-assisted language learning environments shape self-regulation, autonomy, and self-directed behaviors. Language Teaching Research, 0(0), 1-23. https://doi.org/10.1177/13621688261422129

5.Yao, N., Yang, L., & Mutlu, A. K. (2025). Teacher interpersonal behaviours, communication apprehension, self-efficacy and academic engagement in technology-mediated EFL classrooms: A cross-sectional study of Mongolian trilingual learners. European Journal of Education, 61(1), 1-13. https://doi.org/10.1111/ejed.70397

Response: Thank you for your valuable suggestion and for recommending the relevant references. We have carefully read the five papers you suggested and have cited three of them (Derakhshan & Park, 2026a; Derakhshan & Park, 2026b; Yang, 2026) in the revised paper, as they are highly relevant to our discussions on AI assisted language learning, learner emotions, and student engagement, thereby enhancing the engagement with current literature. The other two studies, while academically valuable, focus primarily on teachers’ GenAI literacy, as well as Teacher interpersonal behaviours, communication apprehension, self-efficacy and academic engagement , which differ from the specific focus of the present study on feedback mechanisms and writing development pathways. Therefore, they were not cited in the manuscript. We appreciate your thoughtful guidance.

Comment 8：In the conclusion, the limitations are not well aligned with the directions for future research.

Response：We carefully reviewed the statements regarding limitations and future research directions in the conclusion section and found that the original text indeed failed to adequately address the second limitation (namely, the lack of a deep qualitative analysis of the learner's internalization of feedback). Consequently, we have made targeted revisions. The revised version retains the original focus on extending the study duration, expanding the sample size, and designing syntactic strategies, while also incorporating qualitative research methods, thereby directly addressing the limitation of “lack of deep qualitative analysis.” The current limitations and future research directions are now well aligned.

Response to Reviewer #2：

Reviewer #2: 1. The topic is highly relevant, addressing a critical gap in L2 writing research: the comparative efficacy of traditional teacher feedback and emerging AI-mediated collaborative feedback. The practical implications are clear, especially for application-oriented institutions seeking efficient, scalable feedback models. The theoretical framing within Sociocultural Theory is appropriate, though the potential to contribute to broader debates in SLA (e.g., about mediation, internalization, and the role of technology in scaffolding) could be more explicitly articulated.

Response：We extend our sincere gratitude for your recognition of the relevance of this research topic and the applicability of its theoretical framework. We particularly appreciate your valuable suggestion to clearly articulate the “contribution to broader debates in SLA”. Based on your feedback, we have strategically enhanced the discussion of the broader SLA issues in the introduction and literature review sections of the revised draft, particularly in the theoretical framework and the identification of research gaps. Key revisions include: Regarding mediation and internalization mechanisms, we explicitly state in “Literature Review——Theoretical Framework: Sociocultural Theory and Feedback in Second Language Writing” that “feedback essentially serves as an external scaffold whose value depends on its precise alignment with the learner’s cognitive potential within the ZPD and its gradual withdrawal at appropriate moments, thereby enabling learners to develop independent task completion skills.” We also emphasize the “dialogic” and “contextualized” nature of written feedback, thereby directly incorporating the mediation and internalization processes into our analytical framework.

Regarding the role of technology in scaffolding: In “Literature Review”, we specifically examined the theoretical limitations of the traditional SCT framework, which categorizes technological tools merely as static auxiliary intermediaries, and noted that “with the emergence of generative AI, the scaffolding attribute of AI remains inadequately theorized.” We explicitly defined AI-assisted peer feedback as a “social-technical composite scaffold.” This discussion directly addresses your inquiry into “the role of technology in scaffolding-based teaching.”

In the section on research gaps and contributions, we explicitly state that this study aims to conduct a systematic comparative analysis of the scaffolding effects of teacher feedback versus AI-assisted peer feedback within the SCT framework, thereby providing empirical evidence for the effectiveness of ‘technology-mediated social collaboration scaffolding.’ This approach closely aligns the research contributions with the core theoretical debates in SLA—mediation, internalization, and technological scaffolding.

The aforementioned revisions have provided a clearer and more in-depth elaboration of this study’s potential contribution to the broader issues of SLA. Once again, we extend our gratitude for your meticulous guidance and constructive feedback.

Reviewer #2: 2. The literature review is comprehensive, covering key areas: teacher feedback, peer feedback, AWE, and generative AI feedback. It successfully identifies a research gap—the lack of comparative studies within a unified theoretical framework—and positions the study accordingly. However, the review tends toward description rather than critical synthesis. For instance, while AI-assisted peer feedback is presented as a novel “technologically-intermediary social collaborative scaffold,”

Attachment Submitted filename: Response to Reviewers.docx

# Peer review: Decision Letter 1

**DOI:** 10.1371/journal.pone.0345976.r003

**Authors:** Ali Derakhshan, Ali Derakhshan

8 Jun 2026

Teacher Feedback VS AI-Assisted Peer Feedback in L2 Writing: A Quasi-Experimental Study in a Chinese University

PONE-D-26-08099R1

Dear Dr. Luo,

We’re pleased to inform you that your manuscript has been judged scientifically suitable for publication and will be formally accepted for publication once it meets all outstanding technical requirements.

Within one week, you’ll receive an e-mail detailing the required amendments. When these have been addressed, you’ll receive a formal acceptance letter and your manuscript will be scheduled for publication.

An invoice will be generated when your article is formally accepted. Please note, if your institution has a publishing partnership with PLOS and your article meets the relevant criteria, all or part of your publication costs will be covered. Please make sure your user information is up-to-date by logging into Editorial Manager at Editorial Manager® and clicking the ‘Update My Information' link at the top of the page. For questions related to billing, please contact billing support.

If your institution or institutions have a press office, please notify them about your upcoming paper to help maximize its impact. If they’ll be preparing press materials, please inform our press team as soon as possible -- no later than 48 hours after receiving the formal acceptance. Your manuscript will remain under strict press embargo until 2 pm Eastern Time on the date of publication. For more information, please contact onepress@plos.org.

Kind regards,

Ali Derakhshan

Academic Editor

PLOS One

Additional Editor Comments (optional):

Dear Rong,

Thank you for the revised file. I am happy with the revised file.

Best,

Ali Derakhshan

Reviewers' comments:

Reviewer's Responses to Questions

**Comments to the Author**

1. If the authors have adequately addressed your comments raised in a previous round of review and you feel that this manuscript is now acceptable for publication, you may indicate that here to bypass the “Comments to the Author” section, enter your conflict of interest statement in the “Confidential to Editor” section, and submit your "Accept" recommendation.

Reviewer #1: All comments have been addressed

Reviewer #2: All comments have been addressed

**********

2. Is the manuscript technically sound, and do the data support the conclusions?

The manuscript must describe a technically sound piece of scientific research with data that supports the conclusions. Experiments must have been conducted rigorously, with appropriate controls, replication, and sample sizes. The conclusions must be drawn appropriately based on the data presented.

Reviewer #1: Yes

Reviewer #2: Yes

**********

3. Has the statistical analysis been performed appropriately and rigorously?

Reviewer #1: Yes

Reviewer #2: Yes

**********

4. Have the authors made all data underlying the findings in their manuscript fully available?

The PLOS Data policy requires authors to make all data underlying the findings described in their manuscript fully available without restriction, with rare exception (please refer to the Data Availability Statement in the manuscript PDF file). The data should be provided as part of the manuscript or its supporting information, or deposited to a public repository. For example, in addition to summary statistics, the data points behind means, medians and variance measures should be available. If there are restrictions on publicly sharing data—e.g. participant privacy or use of data from a third party—those must be specified.

Reviewer #1: Yes

Reviewer #2: Yes

**********

5. Is the manuscript presented in an intelligible fashion and written in standard English?

PLOS ONE does not copyedit accepted manuscripts, so the language in submitted articles must be clear, correct, and unambiguous. Any typographical or grammatical errors should be corrected at revision, so please note any specific errors here.

Reviewer #1: Yes

Reviewer #2: Yes

**********

6. Review Comments to the Author

Please use the space provided to explain your answers to the questions above. You may also include additional comments for the author, including concerns about dual publication, research ethics, or publication ethics. (Please upload your review as an attachment if it exceeds 20,000 characters)

Reviewer #1: (No Response)

Reviewer #2: As the authors revised accordingly, I therefore have no further comments. But the response has some format errors, which is not reader friendly.

**********

7. PLOS authors have the option to publish the peer review history of their article (what does this mean?). If published, this will include your full peer review and any attached files.

If you choose “no”, your identity will remain anonymous but your review may still be made public.

**Do you want your identity to be public for this peer review?**  For information about this choice, including consent withdrawal, please see our Privacy Policy.

Reviewer #1: No

Reviewer #2: No

**********

# Editor report: Acceptance letter

**DOI:** 10.1371/journal.pone.0345976.r004

**Authors:** Ali Derakhshan, Ali Derakhshan

PONE-D-26-08099R1

PLOS One

Dear Dr. Luo,

I'm pleased to inform you that your manuscript has been deemed suitable for publication in PLOS One. Congratulations! Your manuscript is now being handed over to our production team.

At this stage, our production department will prepare your paper for publication. This includes ensuring the following:

* All references, tables, and figures are properly cited

* All relevant supporting information is included in the manuscript submission,

* There are no issues that prevent the paper from being properly typeset

You will receive further instructions from the production team, including instructions on how to review your proof when it is ready. Please keep in mind that we are working through a large volume of accepted articles, so please give us a few days to review your paper and let you know the next and final steps.

Lastly, if your institution or institutions have a press office, please let them know about your upcoming paper now to help maximize its impact. If they'll be preparing press materials, please inform our press team within the next 48 hours. Your manuscript will remain under strict press embargo until 2 pm Eastern Time on the date of publication. For more information, please contact onepress@plos.org.

You will receive an invoice from PLOS for your publication fee after your manuscript has reached the completed accept phase. If you receive an email requesting payment before acceptance or for any other service, this may be a phishing scheme. Learn how to identify phishing emails and protect your accounts at https://explore.plos.org/phishing.

If we can help with anything else, please email us at customercare@plos.org.

Thank you for submitting your work to PLOS ONE and supporting open access.

Kind regards,

PLOS ONE Editorial Office Staff

on behalf of

Dr. Ali Derakhshan

Academic Editor

PLOS One
